# Structural basis of broad HIV neutralization by a vaccine-induced cow antibody

**DOI:** 10.1126/sciadv.aba0468

**Published:** 2020-05-27

**Authors:** Robyn L. Stanfield, Zachary T. Berndsen, Ruiqi Huang, Devin Sok, Gabrielle Warner, Jonathan L. Torres, Dennis R. Burton, Andrew B. Ward, Ian A. Wilson, Vaughn V. Smider

**Affiliations:** 1 Department of Integrative Structural and Computational Biology, The Scripps Research Institute, La Jolla, CA 92037, USA.; 2 IAVI, Neutralizing Antibody Center, The Scripps Research Institute, La Jolla, CA 92037, USA.; 3 Scripps Consortium for HIV/AIDS Vaccine Development, The Scripps Research Institute, La Jolla, CA 92037, USA.; 4 Department of Molecular Medicine, The Scripps Research Institute, La Jolla, CA 92037, USA.; 5 Applied Biomedical Science Institute, San Diego, CA 92127, USA.; 6 Taurus Biosciences LLC, San Diego, CA 92127, USA.; 7 Department of Immunology and Microbiology, The Scripps Research Institute, La Jolla, CA 92037, USA.; 8 Ragon Institute of Massachusetts General Hospital, Massachusetts Institute of Technology, and Harvard University, Cambridge, MA 02114, USA.; 9 Skaggs Institute for Chemical Biology, The Scripps Research Institute, La Jolla, CA 92037, USA.

## Abstract

Potent broadly neutralizing antibodies (bnAbs) to HIV have been very challenging to elicit by vaccination in wild-type animals. Here, by x-ray crystallography, cryo–electron microscopy, and site-directed mutagenesis, we structurally and functionally elucidate the mode of binding of a potent bnAb (NC-Cow1) elicited in cows by immunization with the HIV envelope (Env) trimer BG505 SOSIP.664. The exceptionally long (60 residues) third complementarity-determining region of the heavy chain (CDR H3) of NC-Cow1 forms a mini domain (knob) on an extended stalk that navigates through the dense glycan shield on Env to target a small footprint on the gp120 CD4 receptor binding site with no contact of the other CDRs to the rest of the Env trimer. These findings illustrate, in molecular detail, how an unusual vaccine-induced cow bnAb to HIV Env can neutralize with high potency and breadth.

## Teaser

Structure and mutagenesis studies show that a potent vaccine-elicited cow bnAb binds with a compact footprint to HIV gp120 CD4bs.

## INTRODUCTION

The HIV-1 envelope (Env) protein is a heavily glycosylated trimer consisting of heterodimeric gp120 and gp41. For many years, it was thought that the extensive glycosylation coating the surface of the Env trimer served as an impenetrable shield against antibody neutralization (*1*), and before 2009, only a handful of anti-gp120 or anti-gp41 broadly neutralizing antibodies (bnAbs) had been characterized (*2*). However, from 2009, the advent of single B cell approaches to human monoclonal antibody isolation and the availability of suitable donors enabled isolation of many potent bnAbs from the sera of HIV-infected patients (*3*–*5*). Potent bnAbs are found in a few percentage of HIV-infected individuals and do not usually emerge until 2 to 4 years after initial infection (*6*). Perhaps unexpectedly, all exposed surfaces of the HIV trimer have now been shown to be targeted by bnAbs (*7*), with most epitopes containing both protein and carbohydrate components (*8*). A subset of these human bnAbs have very long third complementarity-determining region of heavy chain (CDR H3) regions (*7*) of up to 38 amino acids (especially those targeting the trimer apex) that can pierce through the dense “glycan shield” of HIV Env, contacting both protein and glycan.

About 10% of bovine immunoglobulins contain an “ultralong,” disulfide-rich CDR H3 containing up to 70 amino acids (*9*–*11*). Crystal structures of 12 unliganded bovine Fabs (*10*, *12*, *13*) have shown that the ultralong CDR H3 folds into a highly extended structure with a long β-ribbon stalk supporting an unusual small knob domain that protrudes up to 40 Å above the tips of the other CDR loops. The knob domain folds into three short, anti-parallel β strands, connected by loops that vary in size, amino acid composition, and structure. Nonetheless, the knob domain sequences are extremely variable and include from one to four disulfide bonds, only one of which is spatially conserved. These ultralong CDR H3s are produced during VDJ recombination using the specialized bovine IGHV1-7, IGHD8-2, and IGHJ2-4 gene segments, where an unusually long DH gene codes for 48 amino acids (*14*). These antibodies are also present in fetal tissue and appear to undergo somatic mutation before immunogen exposure (*15*). Many human anti-HIV bnAbs also have relatively long CDR H3s compared to the average human antibody that allow them to access epitopes otherwise occluded by the extensive glycosylation on HIV Env. While immunization trials with the soluble BG505 SOSIP Env construct (*16*) in rabbits and nonhuman primates have so far produced mainly autologous neutralization (*17*), broadly neutralizing sera to HIV were elicited with the same Env construct in cows in a remarkably short period of time (with one cow producing 20% breadth in 42 days and 96% breadth at 381 days) (*18*). The most broadly neutralizing bnAb isolated from these sera, NC-Cow1, had 72% neutralization breadth on a 117-virus panel, with a half-maximal inhibitory concentration (IC_50_) of 0.028 μg/ml (*18*). Low-resolution, negative-stain electron microscopy (EM) studies of two of these antibodies indicated that they contact the CD4 receptor binding site (CD4bs) of the SOSIP trimer with the distal tip of their knob domains (*18*). Given the rapid broadly neutralizing response of bovine antibodies with ultralong CDR H3s, it is important to decipher the structural basis of their HIV Env antigen recognition in molecular detail.

## RESULTS

### X-ray and cryo-EM structure determination

**Fig. 1:**
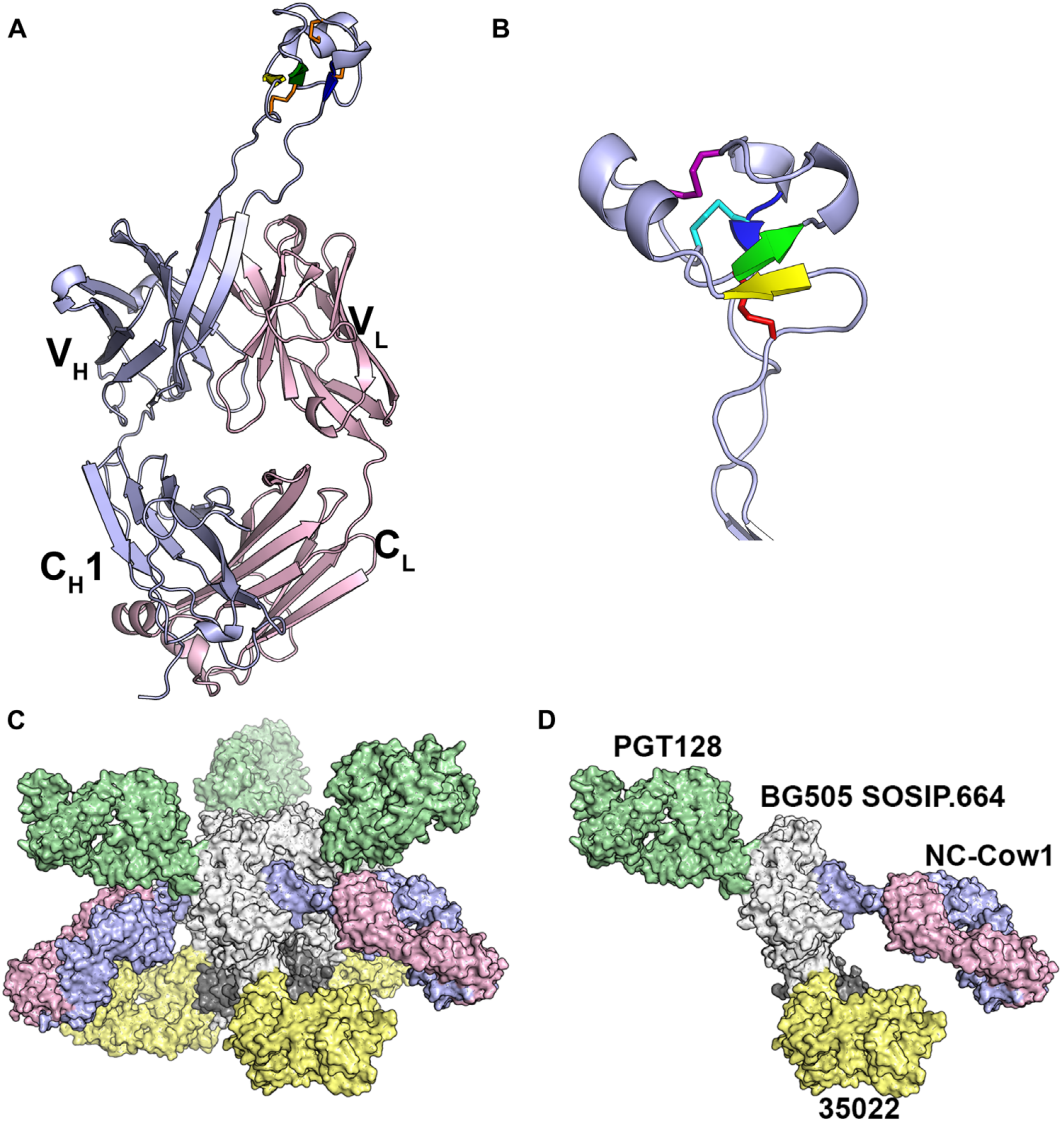
NC-Cow1 crystal structures. (**A**) Crystal structure of unliganded Fab NC-Cow1 at 2.1 Å resolution with heavy chain in light blue and light chain in pink. V_L_, variable region of immunoglobulin light chain. V_H_, variable region of immunoglobulin heavy chain. (**B**) The knob at the tip of the 60-residue CDR H3 contains three β strands (yellow, green, and blue), connected by two loops, with three disulfide bonds (cyan, red, and magenta). (**C**) BG505 SOSIP.664 Env trimer (gray) in complex with three NC-Cow1 Fabs (light chain, pink; heavy chain, blue), three PGT128 Fabs (green), and three 35022 Fabs (yellow). (**D**) The crystallographic asymmetric unit contains one SOSIP protomer (gp120 and gp41) and one each of the NC-Cow1, PGT128, and 35022 Fabs. The PGT128 and 35022 Fabs were added to help obtain diffraction-quality crystals (*19*, *20*).

Crystal structures for Fab NC-Cow1 were determined alone (2.1 Å) and in complex with the BG505 SOSIP.664 Env trimer (4.1 Å) to delineate molecular interactions between a bovine antibody with an ultralong CDR H3 and its antigen (Fig. 1, table S1, and fig. S1). Conventional Kabat numbering is used for the antibody light (L) chain and for heavy (H) chain residues H1-100b and H101 to Fab C terminus, but CDR H3 residues between H100b and H101, encoded by the IGHD8-2 gene segment (and its connection to the J chain), are numbered sequentially with a “D” chain identifier (see fig. S2) as previously described (*12*). As in other bovine Fabs (*10*, *12*, *13*), the ultralong CDR H3 folds into an extended β-ribbon stalk supporting the disulfide-bonded knob domain. In contrast to previous structures, the stalk forms a regular, hydrogen-bonded ribbon only at its base, with the intervening structure between base and knob adopting a more irregular structure that lacks canonical main-chain hydrogen bonds (Fig. 1 and fig. S3). However, several interactions within this stalk section may aid in its stabilization (fig. S3), including a hydrogen bond network between GluD1, ArgD43, and AsnH100 and stacking of the side chains of AsnD39 on HisD42, TyrD40 on LysH100b, and IleD41 on ArgD43. The knob domain begins with a conserved CPED motif (CPDG in the germline) containing a type I β-turn around PEDY, followed by three very short, antiparallel β strands (D6-D8, D23-D25, and D37-D38) with two intervening loops of 14 and 11 residues. Loop 1 forms a single turn of helix, while loop 2 has two small helical turns. The knob has three disulfide bonds with 1-4, 2-5, and 3-6 connectivity (D2-D23, D12-D32, and D21-D37). The knob distal tip also contains several Tyr and Arg residues. Fab NC-Cow1 is similar in overall architecture to other bovine ultralong CDR H3 Fabs (fig. S3, C and D). The knob distal end extends about 35 to 40 Å from the tips of the other CDR loops. Superposition of the Fabs or even of the CDR H3 stalks does not necessarily superimpose the knob domains, as the knobs can adopt different dispositions with respect to the stalk and Fab core.

The NC-Cow1 structure was then determined to 4.1 Å resolution in a quaternary complex with BG505 SOSIP.664, in which human Fabs PGT128 and 35022 were added to facilitate formation of diffraction-quality crystals (*19*, *20*) (Fig. 1). The preformed complex was partially deglycosylated and purified by size exclusion chromatography (SEC) before crystallization. Env glycans in the vicinity of the bound antibodies are usually protected from cleavage by EndoH (*21*). The B values are lower for the Env trimer compared to the antibody components and increase as a function of distance from the center of the trimer so that the distal constant regions of all three Fabs are poorly ordered, as seen in other Fabs (fig. S4) and, thus, not included in the refined structure.

The NC-Cow1 structure with fully glycosylated BG505 SOSIP.664 produced in human embryonic kidney (HEK) 293F cells was further determined by cryo-EM in C3 symmetry to ~4.5 Å (table S2 and fig. S5). The cryo-EM map is consistent with the previous negative-stain reconstruction (*18*), exhibiting well-resolved density for the NC-Cow1 knob domain but poorly resolved density for the remaining Fab structure (fig. S5), indicating flexibility in Fab binding other than the knob domain, similar to the B value distribution observed in the x-ray structure. This observation is further amplified in the final reconstruction because of the use of a tight mask during three-dimensional (3D) refinement that excluded the bulk of the Fab density (fig. S5) so as to improve alignment quality, resolution, and interpretation at the epitope/paratope region.

### X-ray and cryo-EM structure comparison

**Fig. 2:**
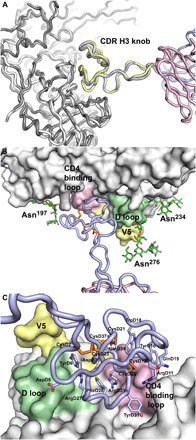
Knob interactions of NC-Cow1 with gp120 in SOSIP Env trimer. (**A**) The x-ray (light blue, heavy chain; light pink, light chain; white, gp120) and cryo-EM (light yellow, heavy chain; dark gray, gp120) structures are superimposed by their gp120 components in the BG505 SOSIP.664 Env trimer and show nearly identical binding of the NC-Cow1 CDR H3 knob. (**B**) The gp120 CD4BL (residues 362 to 374), V5 region (458 to 469), and D loop (275 to 283) in the crystal structure are colored pink, light yellow, and light green, respectively. The glycan (green, carbons; red, oxygens) at Asn^197^ contacts the CDR H3 knob, while some of the sugar moieties in the glycans at Asn^276^ and Asn^234^ have ordered electron density but make little or no contact with the Fab. (**C**) Knob residues contacting gp120 are highlighted with color coding as in (B).

**Fig. 3:**
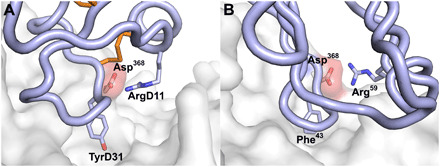
Comparison of binding of NC-Cow1 and CD4 to gp120. The gp120 is shown with a white surface, with the Asp^368^ oxygen atoms colored red and the Asp side chain in stick representation. NC-Cow1 (**A**) mimics the interaction of CD4 (**B**) through insertion of an aromatic residue into the Phe^43^ binding pocket (TyrD31) and through formation of a salt bridge from ArgD11 to gp120 Asp^368^.

The crystal and cryo-EM structures of NC-Cow1 with BG505 SOSIP.664 reveal very similar binding interactions with the distal surface of the knob domain contacting the gp120 CD4bs (Fig. 2). Loops 1 (D9-D22) and 2 (D26-D36) form a jaw-like structure that interacts with the gp120 CD4 binding loop (CD4BL) (Fig. 2A). NC-Cow1 paratope residues include GluD4, AspD5, TyrD6, AsnD9, ArgD11, GlnD14, GlnD15, TyrD16, TrpD18, ArgD27, PheD28, TyrD31, ArgD33, AspD35, and the disulfide between CysD12 and CysD32 (Fig. 2). PheD28 at the paratope geometric center is buried in a shallow pocket and flanked by ArgD27 and ArgD33, while the paratope perimeter is composed largely of charged and polar residues including many Asp, Asn, Glu, and Gln. Although TrpD18 makes only minor contact with gp120, it packs between TyrD16 and GlnD34, possibly helping to stabilize the knob. The antibody-antigen interaction buries ~834 and 768 Å^2^ of molecular surface on gp120 and Fab, respectively (tables S3 and S4). In the crystal structure, strong density is observed for the glycan at gp120 Asn^276^, but only a few contacts from the stalk HisD42 and ArgD43 are made with this glycan (only ~19 Å^2^ of Asn^276^ glycan molecular surface is buried by the Fab). It is possible that the tip of the glycan at Asn^276^ may contact the Fab light chain, but any such interaction is transitory or not stable enough to be observed in the density maps (fig. S1). This glycan would be expected to be protected by the antibody during EndoH deglycosylation if there is contact or close proximity, as we have observed in other deglycosylated Env complexes in the presence of antibodies (*21*). That is borne out in the crystal structure as we see multiple glycan moieties at Asn^276^, where only one sugar (*N*-acetyl glucosamine) would remain if this glycan had been cleaved by EndoH (fig. S1). The glycan at gp120 Asn^197^ is visualized in both the cryo-EM and crystal structures, albeit with a slightly different conformation, and contacts knob GlnD15 and TyrD16 with 115 Å^2^ of buried glycan molecular surface in the crystal structure and 150 Å^2^ in the cryo-EM structure. The glycan composition at Asn^197^ is about 85% oligomannose and 15% complex carbohydrate in BG505 SOSIP.664 made in 293F cells (*22*), while it is primarily oligomannose in material made in 293S cells. Other minor differences between the crystal and cryo-EM structures occur in loops around gp120 residues 364 to 371, 426 to 432, and 459 to 467; however, these differences are likely related to the low resolution of the structure. NC-Cow1 Fab makes two interactions that mimic CD4 receptor binding. TyrD31 inserts into the gp120 CD4 Phe^43^ binding pocket, and ArgD11 makes a salt bridge with gp120 Asp^368^ that emulates the CD4 Arg^59^ to gp120 Asp^368^ interaction (Fig. 3) (*23*). However, NC-Cow1 binding does not trigger the CD4-induced conformational change that forms the bridging sheet in the CD4-bound structure (fig. S6).

While the light chains that pair with ultralong CDR H3 antibodies usually use a single germline V_L_ (variable region of immunoglobulin light chain) gene (V30) and are often relatively invariant in sequence, NC-Cow1 light chain has several mutations from the V30 germline sequence at Kabat positions 1, 2, 3, 19, 47, 60, 86, 91, and 92 (fig. S2). Most of these positions are distant from the CDRs, except for A91P and E92D that are close to the base of CDR H3. However, as these mutations do not appear to change the main-chain conformation, they do not appear critical for binding specificity to BG505 SOSIP.664.

### Site-directed mutagenesis

**Fig. 4:**
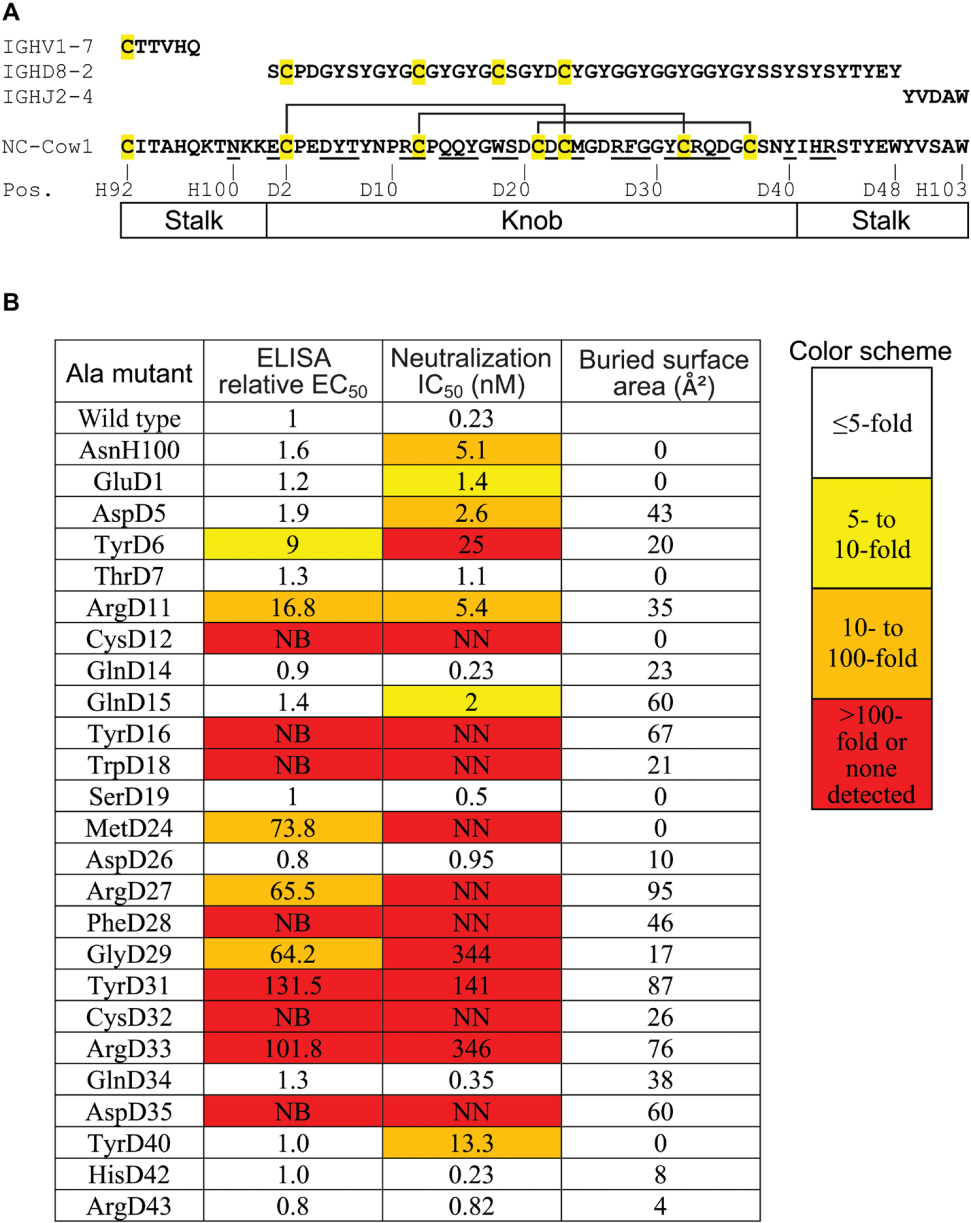
NC-Cow1 Fab alanine mutants affect binding and neutralization. (**A**) Sequence alignment of NC-Cow1 CDR H3 with the germline–encoded IGHV1-7, IGHD8-2, and IGHJ2-4 segments. The numbering scheme for the bovine ultralong CDR H3 region follows reference (*12*). V, D, and J gene usage was determined as described in (*11*) using the IMGT database and BLASTn. Twenty-five residues in the knob and stalk region (underlined) were mutated to alanine. Cysteines are shaded in yellow, and the disulfide pattern is indicated above the sequence. (**B**) Residues that are important for Fab binding and neutralization to BG505 SOSIP.664 trimers. Ratios of half-maximal effective binding concentrations (EC_50_) of each mutant relative to wild-type Fab (set to 1) were calculated. The neutralization potency of each alanine mutant was measured using IC_50_. The EC_50_ and IC_50_ values are shaded yellow, orange, and red according to the color scheme. For the ELISA measurements: NB, no binding activity/curve could be generated; for the neutralization measurements: NN, no neutralization detected. The surface areas buried on gp120 by each Fab residue (as calculated from the crystal structure) are shown on the right.

NC-Cow1 Fab alanine mutants were tested for binding to BG505 SOSIP.664 trimers by enzyme-linked immunosorbent assay (ELISA) and for neutralization on BG505 pseudovirus (Fig. 4 and fig. S7). Mutation of paratope residues TyrD16, TrpD18, PheD28, and AspD35 to alanine completely eliminated both binding and neutralization, as did mutations to disulfide-bonded CysD12 and CysD32, highlighting the importance of the structural integrity of the knob for binding to and neutralizing HIV. TyrD6, ArgD11, ArgD27, TyrD31, and ArgD33 mutations also reduced binding affinity and neutralization potency by ninefold or more. MetD24, which is buried inside the knob, when mutated, impaired Fab binding and neutralization and may play an important structural role in the knob. In addition, GlyD29 is in close contact with the Env trimer with torsion angles only accessible to glycine (φ = 43°, ψ = 48°) and is the *i* + 1 residue in a type I′ turn, so mutation to alanine may alter its structure and possibly result in clashes with the trimer. The stalks AsnH100, GluD1, and ArgD43 form an internal hydrogen bond network, and the TyrD40 and LysH100b side chains pack against each other (fig. S3) with no direct contact with gp120. However, alanine mutants showed no change in ELISA but greater than threefold reduced neutralization potency, indicating that subtle structural changes in the stalk can be detected in neutralization but not in SOSIP binding assays. Similarly, mutation of GlnD15, which interacts with the Asn^197^ glycan, has relatively minor effects in SOSIP ELISA but more pronounced effects in the neutralization assay.

**Fig. 5:**
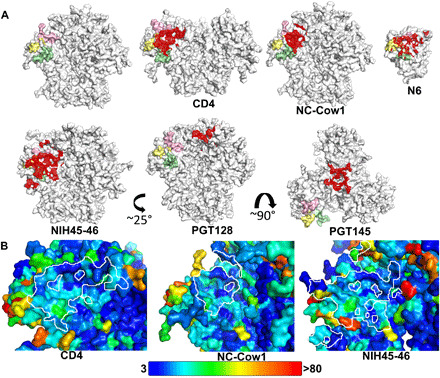
NC-Cow1 binds the CD4 binding site with a compact footprint. (**A**) Highlighted on the BG505 SOSIP.664 trimer (top left) are residues from the CD4BL (362 to 374, pink), V5 loop (458 to 469, pale yellow), and D loop (275 to 283, pale green). The buried molecular surfaces on the trimer or gp120 core are highlighted in red for CD4 (PDB 6CM3), NC-Cow1, human bnAb N6 (PDB 5TE7) (top row), and NIH45-46 (PDB 5D9Q) (bottom left) overlaid onto the key CD4bs elements. With the exception of N6, comparisons were made only to structures determined for Fabs in complex with the BG505 SOSIP trimer. While many structures exist for CD4 binding site Fabs bound to deglycosylated gp120 cores, these may appear to have smaller epitopes because of lack of contact with glycans, missing variable regions, and neighboring trimer subunits. Wild-type N6 has not been determined in complex with this trimer but has been included in the comparison (bound to gp120 core), as it is the most potent and broadly neutralizing CD4 binding site Fab determined to date. The V3 base/N332 glycan and apex binding sites for PGT128 (PDB 5ACO) and PGT145 (PDB 5V8L) (bottom middle and bottom right), respectively, are also shown for comparison. The unbound, CD4-bound, NC-Cow1–bound, and NIH45-46–bound trimers and the N6-bound gp120 core are all in the same orientation, with their gp120 subunits superimposed. The PGT128 trimer is shown rotated ~25° about the vertical trimer axis, and the PGT145-bound trimer is shown rotated ~90° about a horizontal axis. (**B**) The binding footprint for CD4 (left; 6CM3), NC-Cow1 (middle), and NIH45-46 (right; 5D9Q) on the BG505 SOSIP.664 trimer is outlined in white. The SOSIP surface is colored in a rainbow spectrum by the Wu-Kabat variability (*24*) of each residue (see table S3 for numerical values), with conserved regions colored dark blue and highly variable regions colored red. The darkest blue corresponds to Wu-Kabat variability values of 3 or less, while the red corresponds to Wu-Kabat variability value over 80, as shown on the color bar.

Several antibodies isolated alongside NC-Cow1 appear to have evolved from the same germline genes but were less broadly neutralizing on a 12-virus global indicator panel. NC-Cow1 neutralized all 12 isolates with an IC_50_ of 0.44 μg/ml, while NC-Cow7, NC-Cow8, NC-Cow9, and NC-Cow10 neutralized 7, 7, 7, and 8 of 12 viruses with IC_50_’s of 8.6, 2.4, 5.0, and 2.9 μg/ml, respectively; however, on a panel of 24 viruses, NC-Cow1 had far greater neutralization breadth with 22 of 24 strains being neutralized at <4 mg/ml, whereas only 13 of 24 strains were neutralized at this level for NC-Cow7 to NC-Cow9 and 15 of 24 for NC-Cow10. Neutralization of BG505, however, was similarly potent between the antibody variants (*18*). Alignment of their CDR H3 sequences show that some of the residues important for binding and neutralization, such as ArgD11 and ArgD27, are only found in NC-Cow1 (fig. S2C). ArgD11 makes the important salt bridge with gp120 Asp^368^, while ArgD27 makes polar interactions with Asn^280^ and Asn^283^. However, in SOSIP ELISA, a D11R mutation improved binding to BG505 SOSIP.664 substantially for NC-Cow7 but to a much lesser extent for NC-Cow8 (fig. S7), which already binds well; thus, ArgD11 reversion is less important for tight binding of NC-Cow8 but could be involved in neutralization breadth. We compared the CD4bs epitopes from BG505 SOSIP.664 complexes with CD4, NC-Cow1, and human bnAbs NIH45-46 and VRC13 (bound to gp120 core) along with the Wu-Kabat variability (Fig. 5 and table S3) (*24*). Highly conserved gp120 residues that contact CD4 and these antibodies include 366 to 368 from the CD4BL and 473 from the β24-α5 region, indicating, as expected, that Asp^368^ is both highly conserved and generally important for specific ligand interaction with the CD4bs.

### Comparison to human CD4bs antibody structures

Many bnAbs from natural infection target the CD4bs. Most of these CD4bs antibodies belong to the VRC01-like class encoded by the human germline IGHV1-2 family, but some use other V_H_ (variable region of immunoglobulin heavy chain) germline genes including 1-46 (8ANC131), 1-69 (VRC13), 2-23 (VRC16), 3-30 (HJ16) [(*25*) and reviewed in (*7*)], 4-59 (CH103) (*26*), and 1-3 (b12) (*27*). Except for HJ16, most CD4bs antibodies form a salt bridge or hydrogen bond to the highly conserved gp120 Asp^368^, mimicking the CD4 Arg^59^–gp120 Asp^368^ salt bridge thought to be a hotspot for CD4 binding. Some CD4bs antibodies fill the CD4 Phe^43^ binding pocket on gp120 with a single residue (usually aromatic), but this feature does not appear to be critical for neutralization in general, as many bnAbs do not make this interaction. For optimal breadth, CD4bs antibodies must contact the CD4BL while avoiding the hypervariable and highly glycosylated V5 region and either avoid or interact with the conserved glycan Asn^276^ in the D loop. NC-Cow1 achieves its breadth by using a small, compact footprint that avoids the barriers to broad neutralization (Fig. 5 and table S3), along with flexibility in the stalk that may allow for a broader range of approach angles to the virus. The molecular surface area buried on BG505 SOSIP.664 by NC-Cow1 is 834 Å^2^, compared to 1735 and 1065 Å^2^ for bnAbs NIH45-46 [Protein Data Bank (PDB) 5D9Q] and CD4 (PDB 6CM3), respectively. The NC-Cow1 interface size is the smallest that we are aware of, with values ranging from 1310 to 1735 Å^2^ for other Fabs to the CD4bs of similar trimers (table S4). While similar in breadth to CD4bs bnAbs from HIV-infected individuals, NC-Cow1 is not as broad as the exceptional CD4bs antibody N6 (98% breadth) (*28*), although NC-Cow1 has a slightly smaller footprint on gp120 (N6 buries ~1018 to 1036 Å^2^ on gp120 core, but no structure is available for wild-type N6 with Env trimer) and avoids contacting variable residues in V5 (Fig. 5). N6 also avoids the V5 region but makes more contact with conserved residues in the D loop (Fig. 5), suggesting that optimal breadth and potency are a fine balance between avoiding variable residues and making sufficient contact with conserved residues for strong interaction. Notwithstanding, NC-Cow1 was generated by immunization with a single immunogen, and its breadth is quite remarkable.

## DISCUSSION

Antibody binding to antigen is a fundamental advance in vertebrate evolution. In most species, neutralization of foreign microbes by antibodies typically occurs through interaction with a combination of the six CDRs on the antibody heavy and light chains. This binding and recognition mechanism is effective for neutralizing many antigens, but some more challenging epitopes are relatively refractory to classic antibody neutralization. Some species have evolved unusual antibody structures; jawless fish use a leucine-rich repeat scaffold instead of an immunoglobulin domain (*29*), camelids and sharks have smaller antibodies devoid of light chains (*29*), and cows have evolved exceptionally long CDR H3s (*9*, *10*). Cows have recently been found to be unique amongst the species tested in their ability to produce a particularly rapid, robust, and broadly neutralizing response to an HIV immunogen (*18*).

Here, the first atomic structures of a cow antibody-antigen complex reveal only the tip of the CDR H3 knob domain that contacts antigen in contrast with conventional antibodies, which use multiple loops (rare exceptions include anti-influenza virus bnAb C05 where a single CDR H3 loop inserts into the small, shallow hemagglutinin receptor binding site (*30*)). Thus, antigen recognition by an independently folded paratope in just one CDR that extends far from the traditional immunoglobulin surface, as in NC-Cow1, represents a new paradigm for antibody-antigen recognition. Certain somatic mutations in the other CDRs and framework regions likely play a structural role in support of CDR H3.

NC-Cow1 was isolated from a cow immunized with a sequence of the single immunogen BG505 SOSIP.664 Env trimer. The sera exhibited astonishing breadth and potency not previously observed in any other experimental animals immunized with a single or combination of Env immunogens. A previous experiment where llamas were immunized with poorly ordered trimers did result in the isolation of a potent and broadly neutralizing anti-CD4 llama antibody; however, the serum neutralization in this experiment showed relatively poor breadth and potency (*31*). A recent single-molecule fluorescence resonance energy transfer study proposed that native Env trimers favor a putative “state 1” ground state conformation (*32*); however, the “state 2” conformation of BG505 SOSIP.664 found in all x-ray and cryo-EM structures to date (*33*), as well as by double electron-electron resonance spectroscopy (*34*), was indisputably successful in eliciting a potent and broadly neutralizing response to HIV (*18*), suggesting that this immunogen and conformation are highly relevant for vaccine studies.

**Fig. 6:**
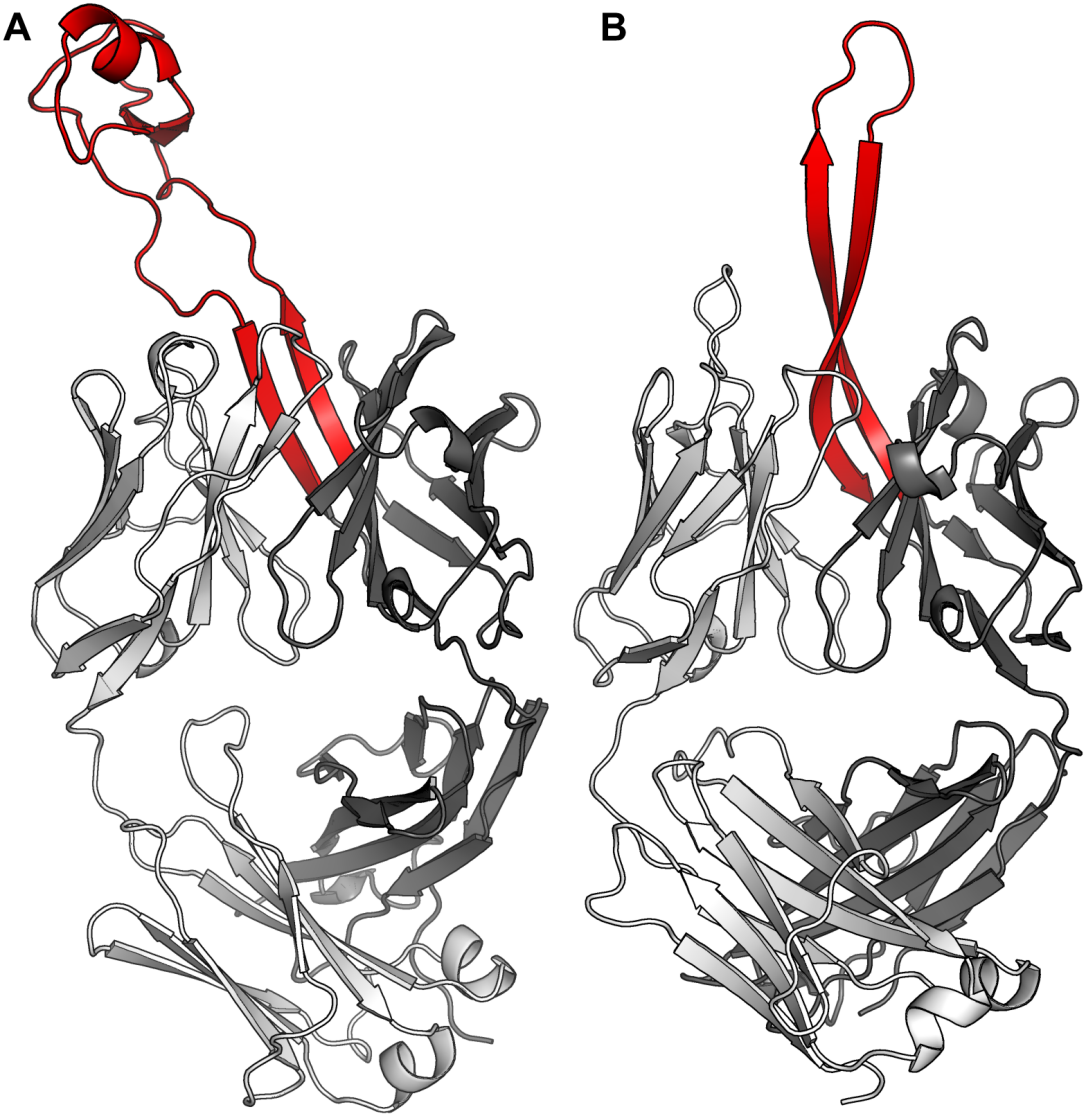
Comparison of NC-Cow1 with human bnAb PGT145. (**A**) NC-Cow1 with light chain in light gray, heavy chain in dark gray, and CDR H3 in red. (**B**) PGT145, colored as in (A), from PDB 3U1S.

Can the information here provide new insights to guide design of an effective human vaccine? It is advantageous to avoid Env variable regions such as V5 and avoid clashes with glycans such as Asn^276^, which is particularly problematic for eliciting neutralizing antibodies to the CD4bs by immunization (*35*, *36*). The long, narrow cow CDR H3 helps navigate past the glycans to contact the underlying CD4bs. CDR H3 (33 residues) of human bnAb PGT145 (H93-H102) (Fig. 6) projects out to almost the same extent as CDR H3 of NC-Cow1 but is a simple β-hairpin, without a knob domain at its tip. PGT145 CDR H3 penetrates the dense glycan shield at the trimer apex, contacting both protein and carbohydrate (*37*) to bury ~1330 Å^2^ on the trimer (Fig. 5), while NC-Cow1 mainly contacts protein using its knob tip. Analysis of germline usage in long human CDR H3s is often quite difficult, and PGT145 CDR H3 and other long human CDR H3s may be derived from extensive N or P additions or might arise from usage of JH6 (which can code for up to 20 residues) or possibly from D-D recombination. The path toward a long CDR H3 is far simpler in cows because of their long, germline–encoded, IGHD8-2 gene, resulting in cows being preloaded with a subset of ultralong CDR H3 antibodies (*14*). For the CD4bs epitope, strong biases in the human germlines selected may suppress selection of long CDR H3s as the primary mode of recognition (*38*). However, while human IGHV1-2 or 1-46 germline antibodies mainly recognize the CD4bs through CDR H2, other bnAbs use other germline genes and primarily use their CDR H3 loops [e.g., VRC13 (23 amino acids), VRC16 (22 amino acids), HJ16 (21 amino acids), and CH103 (15 amino acids)]. Disulfides have also been found in human anti-HIV antibody H3 loops, such as in the CD4bs VRC01 antibody lineage, where VRC01 contains one disulfide connecting H3 to H1 (*39*), VRC03 and VRC06 have one disulfide within H3, and VRC08 has two intra-H3 disulfides (*40*). While VRC08 recognizes CD4bs mainly with CDR H2, its 25 amino acid H3 provides additional contacts to gp120 and neighboring monomer. CAP256-VRC26 lineage bnAbs (targeting V1/V2 apex) also contain one intra-H3 disulfide in their 37– to 38–amino acid CDR H3s (*41*).

Our structural findings also suggest that cows may be particularly useful for vaccine testing and optimization. Engineered vaccines could be rapidly tested for efficacy in cows to validate epitope presentation and construct integrity. The isolated cow knob domains might also be engineered as small protein inhibitors of antigens of interest. Lastly, in an emerging virus outbreak, rapid generation of bovine sera may help in patient treatment. In this regard, bovine species live alongside humans throughout the world and, unlike other model species, should be readily available for immunization trials at or near locations of a viral outbreak. A better understanding of the bovine immune system may also aid in design of vaccines or treatments for cows themselves, as cattle health has substantial impact on worldwide agriculture, or for zoonotic diseases like *Brucella*, Q and Rift Valley fever, *Escherichia coli*, salmonella, listeria, and anthrax (*42*). As most human pathogens are zoonotic, with ungulates being one of the most common hosts [supporting more than 250 human pathogen species (*43*)], in-depth dissection of nonhuman immune systems like cows may ultimately play an important role in control of global infectious disease.

## MATERIALS AND METHODS

### Protein expression and purification

Fabs were expressed by transient cotransfection of 293 FreeStyle cells (Invitrogen), with plasmids containing either the light (V_L_-C_L_) or heavy chain (V_H_-C_H_1). The bovine Fab NC-Cow1 plasmids were designed with bovine variable and human immunoglobulin G1 (IgG1) constant regions, while Fabs 35022 and PGT128 were fully human. Cells were shaken at 37° for 6 days with 8% CO_2_. The medium was clarified by centrifugation at 4000 rpm for 20 min, followed by filtration with a 0.22-μm filter. The medium was then loaded onto a CaptureSelect CH1-XL column (Thermo Fisher Scientific) at 1 ml/min, followed by washing of the column with 5 column volumes of phosphate-buffered saline (PBS) and elution with 0.05 M acetic acid (pH 4) into fractions containing 1 M tris (pH 9). Eluted Fab was further purified by SEC on a Superdex 75 16/60 column with running buffer 0.02 M tris and 0.15 M NaCl (pH 8).

The BG505 SOSIP.664 used for crystallography was coexpressed with furin by transient transfection of 293S FreeStyle cells, with incubation and medium clarification as for the Fabs following a published protocol (*44*). The clarified medium was loaded onto an IgG 2G12 affinity column and eluted with 3 M MgCl_2_. SOSIP was further purified by SEC on a Superdex 200 16/60 column with running buffer 0.02 M tris and 0.15 M NaCl (pH 8). SOSIP was mixed with a 20% excess each of Fabs NC-Cow1, 35022, and PGT128 and incubated for 3 hours at 4°C. This mixture was partially deglycosylated with EndoH for 45 min at 37°C and immediately loaded onto a Superdex 200 16/60 column for purification of the complex. The recombinant BG505 SOSIP.664 for EM was expressed and purified from HEK293F cells using a 2G12 antibody affinity column, followed by SEC. Purified trimers were then mixed with 6 M excess purified NC-Cow1 Fab overnight at 4°C, followed by an additional round of purification with SEC.

### Crystallization

Fab NC-Cow1 (25 mg/ml) and the BG505 SOSIP.664-Fab NC-Cow1-Fab 35022-Fab PGT128 quaternary complex (15 mg/ml) were both screened for crystallization on our robotic Rigaku CrystalMation system at Scripps against 384 different conditions at both 4° and 20°C. The NC-Cow1 crystal used for data collection was grown at 4°C with a precipitant of 0.1 M sodium cacodylate, 40% methyl-pentanediol, and 5% PEG-8000 (polyethylene glycol, molecular weight 800) (pH 6.5). SOSIP-NC-Cow1-35022-PGT128 crystallized in several crystallization conditions, all with low–molecular weight PEG in the precipitant mix, but most of these crystals diffracted x-rays very poorly. The crystal used for data collection was grown in a 24-well Cryschem plate (Hampton Research) at 20°C with precipitant of 0.2 M sodium citrate, 0.1 M tris, and 30% PEG-400 (pH 8.6).

### Data collection, structure solution, and refinement

Data were collected for Fab NC-Cow1 at Advanced Photon Source (APS) beamline 23-ID-B with an EIGER 16M detector and for the quaternary complex at APS beamline 23-ID-D with a PILATUS 6M detector. Data for the unliganded Fab and quaternary complex were processed with XDS (*45*) and HKL-2000 (*46*), respectively. The NC-Cow1 structure was determined by the molecular replacement method using Phaser (*47*) and bovine Fab A01 (PDB 5ILT) as a model. The structure was rebuilt with Coot (*48*), refined with Phenix.refine (*49*) to a final resolution of 2.1 Å, with *R* = 20.2% and *R*_free_ = 23.8% (table S1) and one Fab in the asymmetric unit. The complex structure was determined by molecular replacement, using as models the higher-resolution structures for unliganded Fabs NC-Cow1 (2.1 Å), PGT128 (PDB 3TV3; 1.29 Å), 35022 (PDB 4TOY; 1.55 Å), and BG505 SOSIP.664 from the complex with 35022 and a PGT124 precursor (PDB 5CEZ; 3.0 Å). The structure was refined with Phenix.refine, applying restraints to the initial model, secondary structure restraints, and group B values. The final model extends to a resolution of 4.1 Å with *R*_work_ = 33.3% and *R*_free_ = 34.5% (table S1) with one-third of the trimeric assembly in the asymmetric unit. The constant regions of the Fabs were present but with very weak electron density and were not included in the deposited PDB file. (*R*_work_ and *R*_free_ values with the constant domains included were 33.6 and 34.9%, respectively). Coordinates and structure factors have been deposited into the PDB with codes 6OO0 (native NC-Cow1) and 6OPA (quaternary complex). Representative electron density for the Fab CDR H3 knob can be found in fig. S1.

### EM data collection and processing

Three microliters of purified NC-Cow1-BG505 SOSIP.664 complex at ~6 mg/ml in 1× tris-buffered saline (TBS) was mixed with 1 μl of 0.24 mM *n*-Dodecyl β-D-maltoside and then pipetted onto a 2 by 2 C-Flat holey carbon grid and plunge-frozen with a Vitrobot Mark IV. Imaging was performed on an FEI Talos Arctica operating at 200 keV, with a Gatan K2 direct electron and final calibrated pixel size of 1.15 Å per pixel. Movie micrographs (1506) were collected with a dose rate of 8.44 electrons pixel^−1^ s^−1^ for a total dose of 64 e^−^/Å^2^. Each movie was collected over 10 s and composed of 40 ms by 250 ms frames, which were subsequently aligned and dose-weighted with MotionCor2 (*50*). The contrast transfer function (CTF) parameters were determined for each aligned micrograph with GCTF (*51*), and all subsequent single-particle processing was performed with Electron Microscopy Hole Punch (EMHP) (*52*) and Relion/2.1 (*53*). Briefly, particles were selected automatically from aligned micrographs using template picking in Relion with a Gaussian disk. Micrographs containing carbon edges were masked with EMHP, and raw particle picks were filtered before being extracted and binned four times. Two rounds of 2D classification followed by subset selection were performed followed by recentering and reextraction of unbinned clean particles. One round of 3D autorefinement using C3 symmetry was then performed against an initial model of BG505 SOSIP without any Fabs bound. The resulting map was used to make tight masks that excluded the bulk of the Fab density for further refinement and classification. 3D classification with restricted sampling was then performed to sort out the best particles, followed by subset selection and a final round of 3D autorefinement with a total of 21,427 particles. The final C3 symmetric reconstruction was sharpened with a B factor of −227 Å^2^ using the same tight mask used during refinement. The global map resolution was reported as 4.5 Å at which the gold standard Fourier shell correlation is equal to 0.143 (fig. S5).

### EM model building

The model of BG505 SOSIP.664 in complex with NC-Cow1 Fab was built as follows: First, homology models of BG505 SOSIP.664 gp120 and gp41 were generated with SWISS-MODEL using a previously published crystal structure (PDB 5CEZ) as a template that included the full sequence. The homology models and crystal structure of NC-Cow1 were then fit into the cryo-EM map with UCSF Chimera (*54*) and combined into a single PDB file. N-linked glycans were built at all visible glycosylation sites with the Coot software package (*48*), and this model was then relaxed with C3 symmetry in Rosetta (*55*), “asking for” 300 models. Each model was scored with MolProbity (*56*) and EMRinger (*57*), and the one with the best overall score was selected. Protein residues outside of clear map density were deleted, and manual adjustments to the structure were made with Coot if necessary, followed by a final round of Rosetta relax. The final protein model was validated using MolProbity and EMRinger, and N-linked glycans were validated with Privateer (*58*).

### Analysis of Env sequence variability

Sequence variability in Fig. 5B and table S3 was calculated by the method of Wu and Kabat (*24*) as (*N* × *k*)/*n*, where *N* is the total number of sequences in alignment, *k* is the number of different amino acid types at given position, and *n* is the number of times the most common amino acid is present at that position. A variability of 1.0 means that the residue is 100% conserved.

### Gene annotation

V, D, and J gene usage was determined as described in (*11*) using the IMGT database and BLASTn.

### Analysis of Fab-gp120 interface sizes

Buried surface areas for Fab-gp120 interfaces were calculated with the program MS (*59*) using a 1.7 Å probe radius. For consistency, we tried to use similar structures for comparison, i.e., those where Fabs were complexed to BG505 SOSIP.664 Env trimers; however, interface sizes for Fabs bound to other types of gp120 constructs are listed in table S4 for comparison.

### Quantification of Fab in cell culture supernatant using ELISA

Fab mutants were generated using the QuikChange Lightning Site-Directed Mutagenesis Kit (Agilent) and expressed in 293 FreeStyle cells (Invitrogen), as described above. After collection of cell culture supernatant and filtration through a 0.45-μm filter, Fab mutants in supernatant were quantified using ELISA, as described below. ELISA plates were coated with (50 μl per well) streptavidin (1 μg/ml) at 4°C overnight. Plates were washed three times with 1× TBS + 0.1% Tween 20 (TBST) and blocked with 1% bovine serum albumin (BSA) in 1× TBST at room temperature for 1 hour. CaptureSelect biotin anti–IgG-C_H_1 conjugate (2 μg/ml; 50 μl per well) (Thermo Fisher Scientific) was added to the plates and incubated at room temperature for 1 hour. Plates were washed four times with 1× TBST. Purified bovine Fab wild-type and alanine variants in supernatant were serially diluted in 1% BSA in 1× TBST and added to the wells, and plates were incubated at room temperature for 1 hour. Plates were washed four times with 1× TBST, and goat anti-human lambda conjugated to horseradish peroxidase (HRP; diluted 1:5000 in 1× TBST containing 1% BSA; SouthernBiotech) was added to the wells. Plates were incubated at room temperature for 30 min and washed five times with 1× TBST. Plates were developed by adding 50 μl of 3,3′,5,5′-tetramethylbenzidine (TMB) substrate solution (Thermo Fisher Scientific) per well and then incubated at room temperature for 3 min. The HRP-TMB reaction was stopped by adding 50 μl of 0.5M sulfuric acid per well. The optical density at 450 nm was read on an ultraviolet-visible microplate reader (SpectraMax M2, Molecular Devices). Standard curves and sample concentrations were plotted and calculated using SoftMax Pro (Molecular Devices LLC, San Jose, CA).

### Antigen-binding direct ELISA

ELISA plates were coated with (50 μl per well) soluble BG505 SOSIP trimer (0.2 or 2 ng/μl) at 4°C in 1× PBS (pH 7.4) overnight. Plates were washed three times with 1× TBST and blocked with 1% BSA in 1× TBST at room temperature for 1 hour. Serially diluted Fab variants were added to the wells, and plates were incubated at room temperature for 1 hour. Plates were washed four times with 1× TBST, and then, goat anti-human lambda HRP (diluted 1:5000 in 1× TBST containing 1% BSA; SouthernBiotech) was added to the wells. Plates were incubated at room temperature for 30 min and washed five times with 1× TBST. Plates were developed by adding 50 μl of TMB substrate solution (Thermo Fisher Scientific) per well and incubated at room temperature for 3 min. The HRP-TMB reaction was stopped by adding 50 μl of 1.0 N sulfuric acid per well. The optical density at 450 nm was read on a microplate reader (SpectraMax M2, Molecular Devices). Antigen-binding curves and half-maximal effective concentration (EC_50_) values were generated and calculated using four-parameter logistic regression in GraphPad Prism 8 (GraphPad Software Inc., San Diego, CA). Binding to BG505 SOSIP trimers for each Fab mutant was compared to wild-type Fab side by side on the same ELISA plate (fig. S7).

### Pseudovirus neutralization assays

Plasmids encoding HIV Env were cotransfected into HEK293T cells (American Type Culture Collection) with pSG3ΔEnv, an Env-deficient genomic backbone plasmid, in a 1:2 ratio using X-tremeGENE HP (Roche) as transfection reagent. Cell culture supernatants were harvested 3 days after transfection and sterile-filtered through a 0.22-μm filter. Neutralizing activity was measured by incubating monoclonal antibodies or sera with replication-incompetent pseudovirus for 1 hour at 37°C before transferring onto TZM-bl target cells (aidsreagent.org), as described previously (*60*).

## Supplementary Material

aba0468_SM.pdf

## SUPPLEMENTARY MATERIALS

Supplementary material for this article is available at http://advances.sciencemag.org/cgi/content/full/6/22/eaba0468/DC1

## Appendix

View/request a protocol for this paper from *Bio-protocol*.
