# Supplementary material for: Structural basis of broad HIV neutralization by a vaccine-induced cow antibody
Source: Sci Adv. 2020 May 27;6(22):eaba0468. doi: 10.1126/sciadv.aba0468 (PMC7253169; doi:10.1126/sciadv.aba0468)
Supplement: aba0468_SM.pdf [file aba0468_SM.pdf]

Science Advances
AAAS

advances.sciencemag.org/cgi/content/full/6/22/eaba0468/DC1

Supplementary Materials for

Structural basis of broad HIV neutralization by a vaccine-induced cow antibody

Robyn L. Stanfield, Zachary T. Berndsen, Ruiqi Huang, Devin Sok, Gabrielle Warner, Jonathan L. Torres, Dennis R. Burton, Andrew B. Ward*, Ian A. Wilson*, Vaughn V. Smider*

*Corresponding author. Email: andrew@scripps.edu (A.B.W.); wilson@scripps.edu (I.A.W.); vvsmider@scripps.edu (V.V.S.)

Published 27 May 2020, *Sci. Adv.* **6**, eaba0468 (2020)
DOI: 10.1126/sciadv.aba0468

This PDF file includes:

Figs. S1 to S7
Tables S1 to S4
References

Supplementary Materials

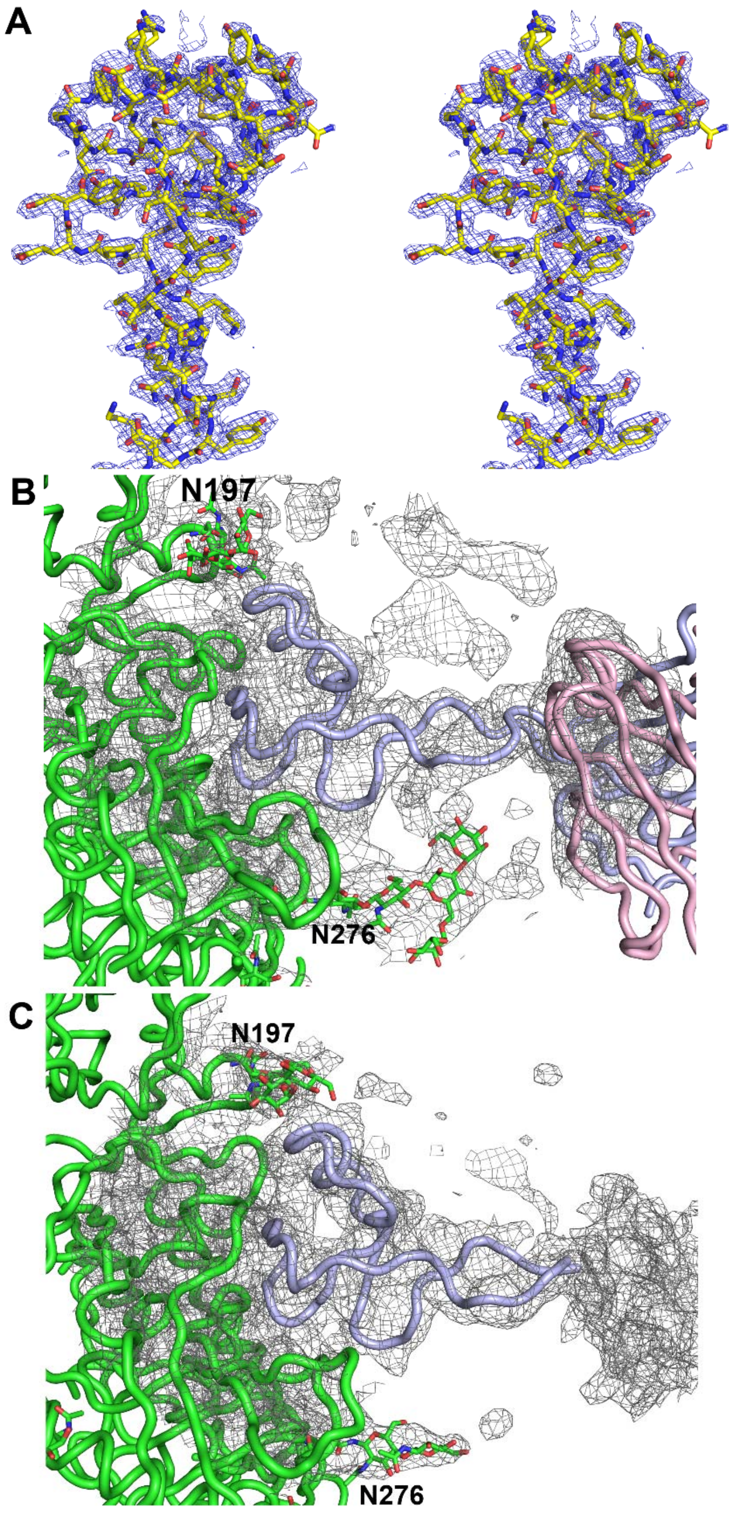

Fig. S1. Density for CDR H3 knob domain in the x-ray and cryo-EM structures. (A) The knob domain (top) attached to the stalk region from the unliganded NC-Cow 1 crystal structure (yellow backbone) with electron density from a 2Fo-Fc map contoured at 1σ (blue mesh). **(B)** The same region is shown

for the complex of NC-Cow1 (blue heavy chain and pink light chain) with BG505 SOSIP.664 (green) with density from a 2Fo-Fc map contoured at 2σ. The glycans at Asn197 and Asn276 are shown as sticks. **(C)** CryoEM density (6σ) is shown for the equivalent region as in (B). In both panels B and C, ordered density from the glycan at Asn276 does not contact the light chain of the Fab; however it is possible that the tip of the glycan might make some weak or transitory contacts that are not observed in the density maps.

```
A
                                L1                     L2
λ chain                     -----------            -----
SYELTQPSSVSGSLGQRVSVTCSGSSSNVGNGYVSWYQLIPGSAPRTIIYGDTSRASGVPERFSGSRSGNTATLTISSLQAEDEADFF
1       11        20      27ab 30        40        50        60        70        80
       L3
  ----------
CASPDDSSSNAVFGSGTTLTVLGQPK
 90   95ab  100   106a 110
                                H1                        H2
Heavy chain                  ---------                 -------
QVQLRESGPSLMKPSQTLSLTCTVSGSSLNDKSVGWVRQAPGKALQWLGSVDTSGNTDYNPGLKSRLSITKDNSKSRISLTVTGMTTEDSATY
1       10        20        30         40        50        60        70         82abc      90
                                H3
  ---------------------------------------------------------------
YCITAHQKTNKKECPEDYTYNPRCPQQYGWSDCDCMGDRFGGYCRQDGCSNYIHRSTYEWYVSAWGQGLL
       100ab      D10       D20       D30       D40        101
```

```
B   Heavy Chain

IGHV1-7            1 QVQLRESGPSLVKPSQTLSLTCTASGFSLSDKAVGWVRQAPGKALEWLGG     50
                       |||||||||||:|||||||||||.||.||:||:||||||||||||:|||.
NC-Cow1_HC         1 QVQLRESGPSLMKPSQTLSLTCTVSGSSLNDKSVGWVRQAPGKALQWLGS     50


IGHV1-7           51 IDTGGSTGYNPGLKSRLSITKDNSKSQVSLSVSSVTTEDSATYYCTTVHQ    100
                       :||.|:|.||||||||||||||||||::||:|:.:||||||||||.|.||
NC-Cow1_HC        51 VDTSGNTDYNPGLKSRLSITKDNSKSRISLTVTGMTTEDSATYYCITAHQ    100


    Light Chain

IGLV30             1 QAVLTQPSSVSGSLGQRVSITCSGSSSNVGNGYVSWYQLIPGSAPRTLIY     50
                       ...||||||||||||||||:|||||||||||||||||||||||||||:||
NC-Cow1_LC         1 SYELTQPSSVSGSLGQRVSVTCSGSSSNVGNGYVSWYQLIPGSAPRTIIY     50

IGLV30            51 GDTSRASGVPDRFSGSRSGNTATLTISSLQAEDEADYFCASAEDSSSNA      99
                       ||||||||||:|||||||||||||||||||||||||:||||.:||||||
NC-Cow1_LC        51 GDTSRASGVPERFSGSRSGNTATLTISSLQAEDEADFFCASPDDSSSNA      99
```

```
                          D1        10        20        30        40        50
C   Germline    CTTVHQ      SCPDGYSYGYGCGYGYGCSGYDCYGYGGYGGYGGYGYSSYSYSYTYEYYVDAW
    NC-Cow1     CITAHQKTNKKECPEDYTYNPRCPQQYGWSDCDCMGDRFGGYCRQDGCSNYIHRSTYEWYVSAW
    NC-Cow7     -T-V---AY--V--D--SS--D-VRL----H----R-S---W--A----STVEIGP-----N--
    NC-Cow10    -T-V-----E-D---Y-S---D--RR----N----A-K---W--H----D-ADMT-D----D--
    NC-Cow8     -T-VY---T--D---Y-----D-ARR------E--A-K------H---ATNTV------HLD--
    NC-Cow9     -T-VY---T--D---Y-----D-ARR------E--A-K------H---ATNTV------HLD--
```

Fig. S2. Sequences for Fab NC-Cow1. (A) Light and heavy variable chain sequence of NC-Cow1 with numbering on the bottom (*1*), and CDR regions on top. **(B)** Alignment of heavy and light chain from NC-Cow1 with cow IGHV1-7 and IGLV30 germline V genes in sequential numbering. **(C)** Sequence alignment of CDR H3 from bnAb NC-Cow1 with germline H3 and with CDR H3 from less broadly neutralizing antibodies NC-Cow7, 8, and 9 and 10. Residues important for binding to the CD4bs in ELISA are highlighted in yellow, orange or red as in Fig. 4B. The germline gene segments from IGVH1-7, IGHD8-2 and IGHJ2-4 are highlighted in gray, green and cyan, respectively.

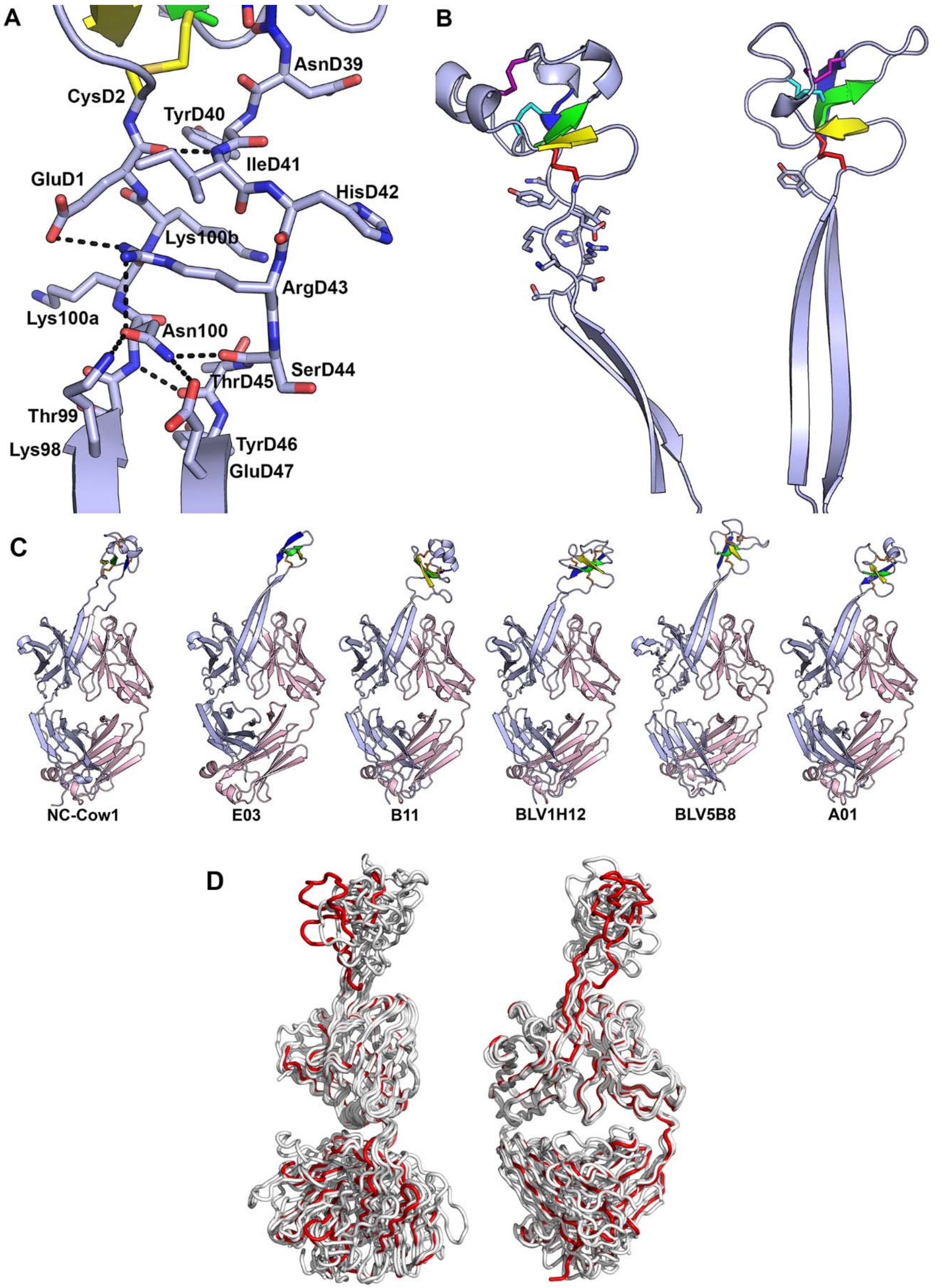

Fig. S3. The NC-Cow1 stalk region. (A) The stalk region of NC-Cow1 has less β-ribbon secondary structure than stalks of previously determined bovine Fabs. However, multiple hydrogen bonds (black dashes) and side-chain stacking appear to stabilize the NC-Cow1 stalk. This analysis and image are based on the unliganded Fab structure. **(B)**

Comparison of the NC-Cow1 stalk (left) with the stalk from Fab BLV1H12 (right, PDB 4K3D). **(C)** Comparison of NC-Cow1 with other bovine ultralong CDR H3 Fabs, with the heavy and light chains shown in light blue and pink and NC-Cow1 on the far left. NC-Cow1 has the most irregular stalk region, with only a short stretch of canonical β-strand interactions in the β-ribbon at the base of its stalk. Structures from PDB codes PDB 5IJV (E03), 5IHU (B11), 4K3D (BLV1H12), 4K3E (BLV5B8), 5ILT(A01). The identity of the antigens for the other Fabs has not been unambiguously proven. (D) Superposition of NC-Cow1 (red) with previous structures of bovine ultralong CDR H3 Fabs E03, B11, BLV1H12, BLV5D8 and A01 (white and gray; PDB 5IJV, 5IHU, 4K3D, 4K3E, 5ILT) shows differences in the relative orientations of the knob domains to the main body of the Fab likely due at least in part to some flexibility in the stalk region. The two views are rotated by approximately 180° around a vertical axis.

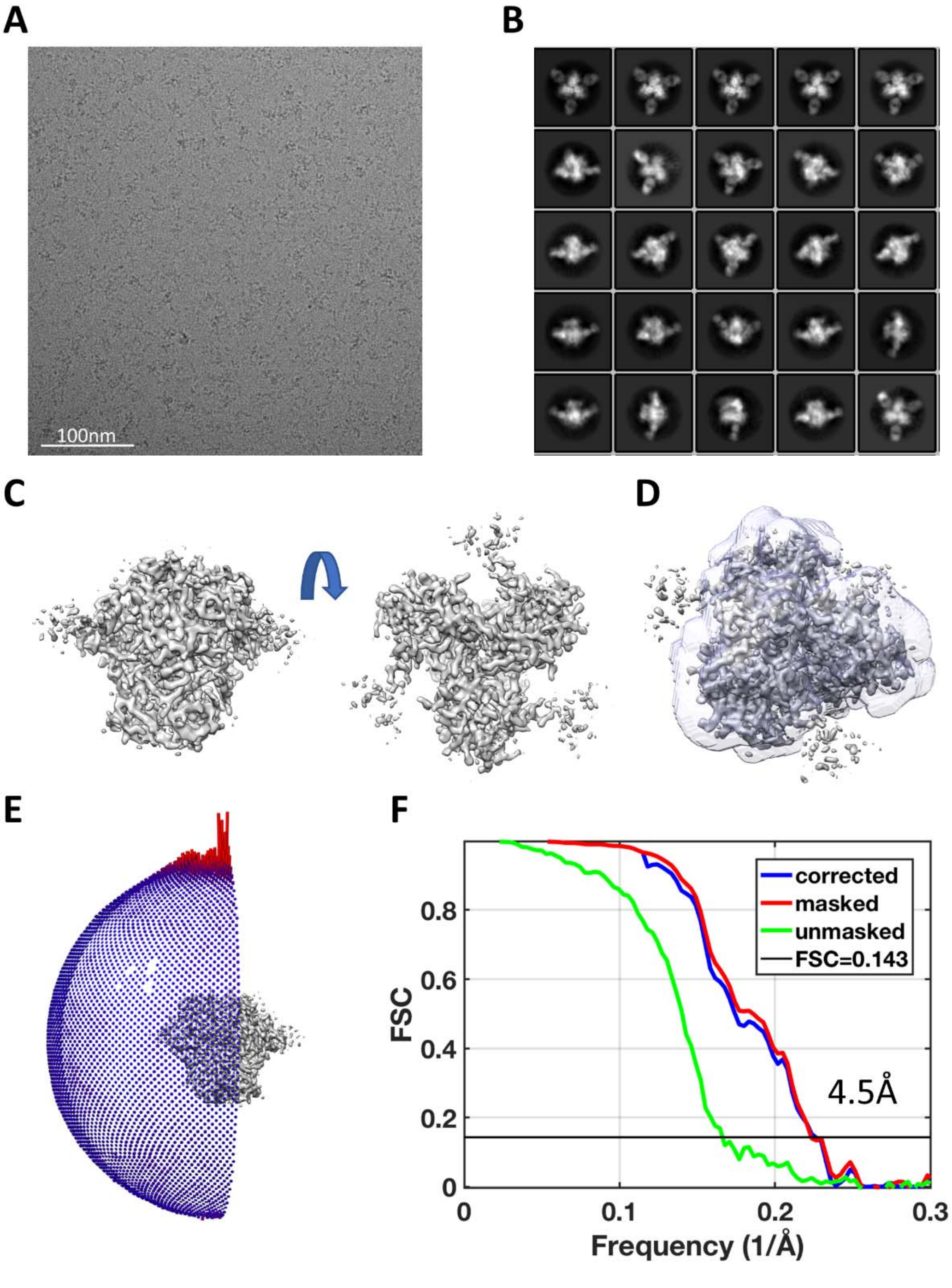

Fig. S4. Single particle cryo-EM reconstruction of BG505 SOSIP.664 in complex with NC-Cow1 Fab. Representative raw micrograph **(A)** and 2D class averages **(B)**. Side and top views of the sharpened EM density map (**C)** and tight 3D mask excluding most of the Fab density used during refinement **(D)**. The Fab density is weaker further from the Env binding site indicating some flexibility between the CDR H3 stalk and the rest of the Fab. The 3D angular distribution histogram (**E**) and Fourier shell correlation plot **(F)** showing the resolution at FSC = 0.143 are illustrated.

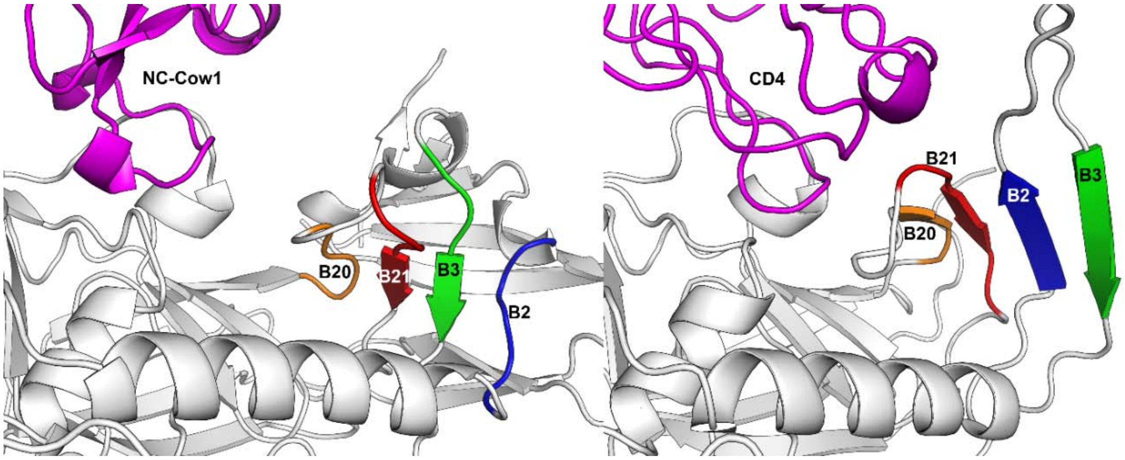

Fig. S5. Comparison of the gp120 bridging sheet in the NC-Cow1-SOSIP and CD4-gp120 complexes. NC-Cow1 binding to the BG505 SOSIP trimer (left) does not induce rearrangement of the bridging sheet region observed in gp120 upon CD4 receptor binding (PDB 2NXY) (right).

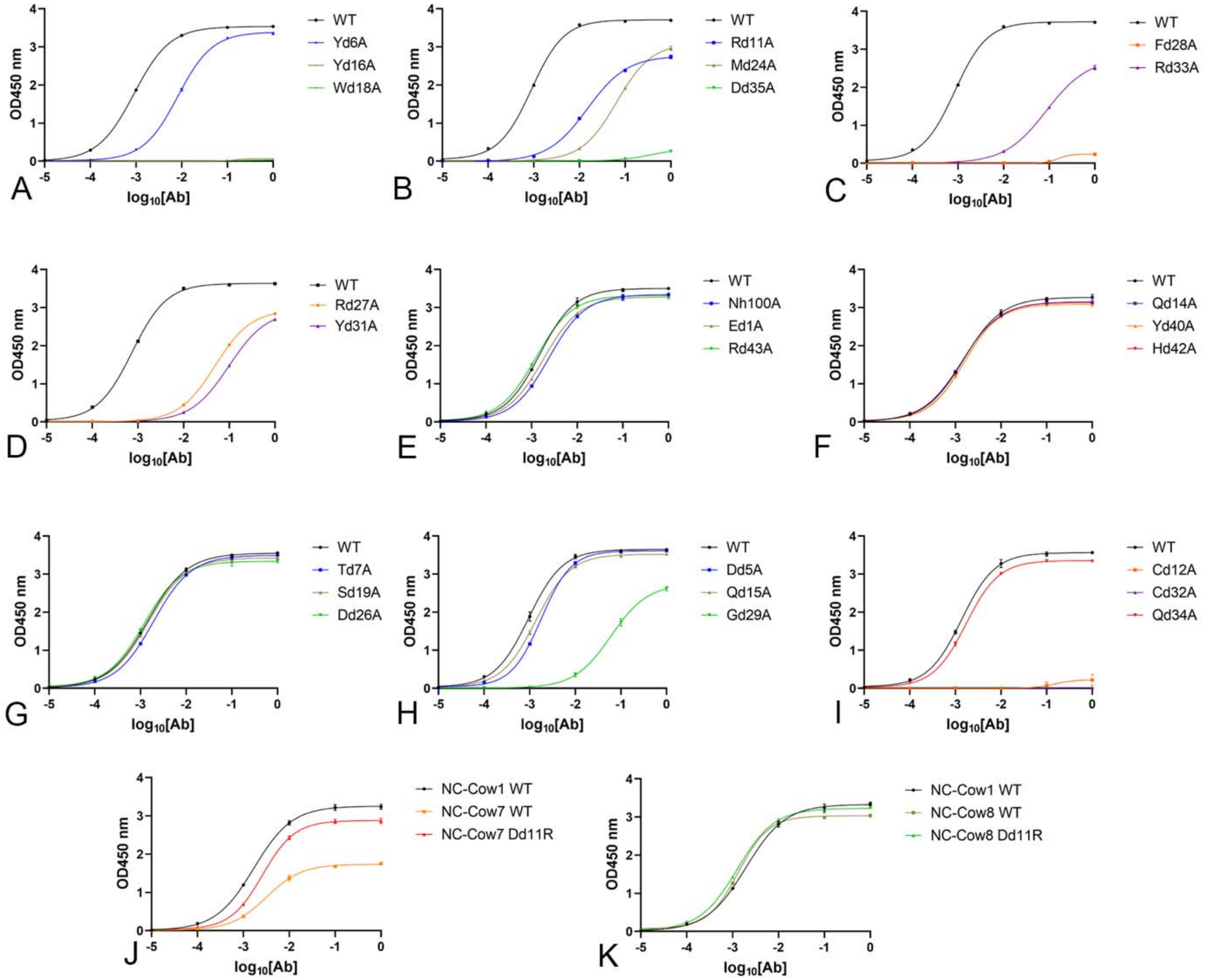

Fig. S6. ELISA binding data. (**A-I**) WT and Ala mutants of Fab NC-Cow1 binding to the BG505 SOSIP.664 trimer**.** Binding curves from ELISA showing the effect of mutating stalk and knob residues on Fab NC-Cow1 with the BG505 SOSIP.664 trimer. Each alanine mutant was compared to the wild-type Fab side-by-side on the same ELISA plate. For Fab numbering, please see fig. S2. (**J-K**) WT NC-Cow1 and WT and Dd11R mutants of IgG NC-Cow7 and NC-Cow8 binding to BG505 SOSIP.664 trimer. Binding curves from ELISA showing the effect of a Dd11R mutation on IgG NC-Cow7 and NC-Cow8 with the BG505 SOSIP.664 trimer. Each mutant was compared to the wild-type IgG and WT NC-Cow1 IgG side-by-side on the same ELISA plate. For IgG numbering, please see fig. S2.

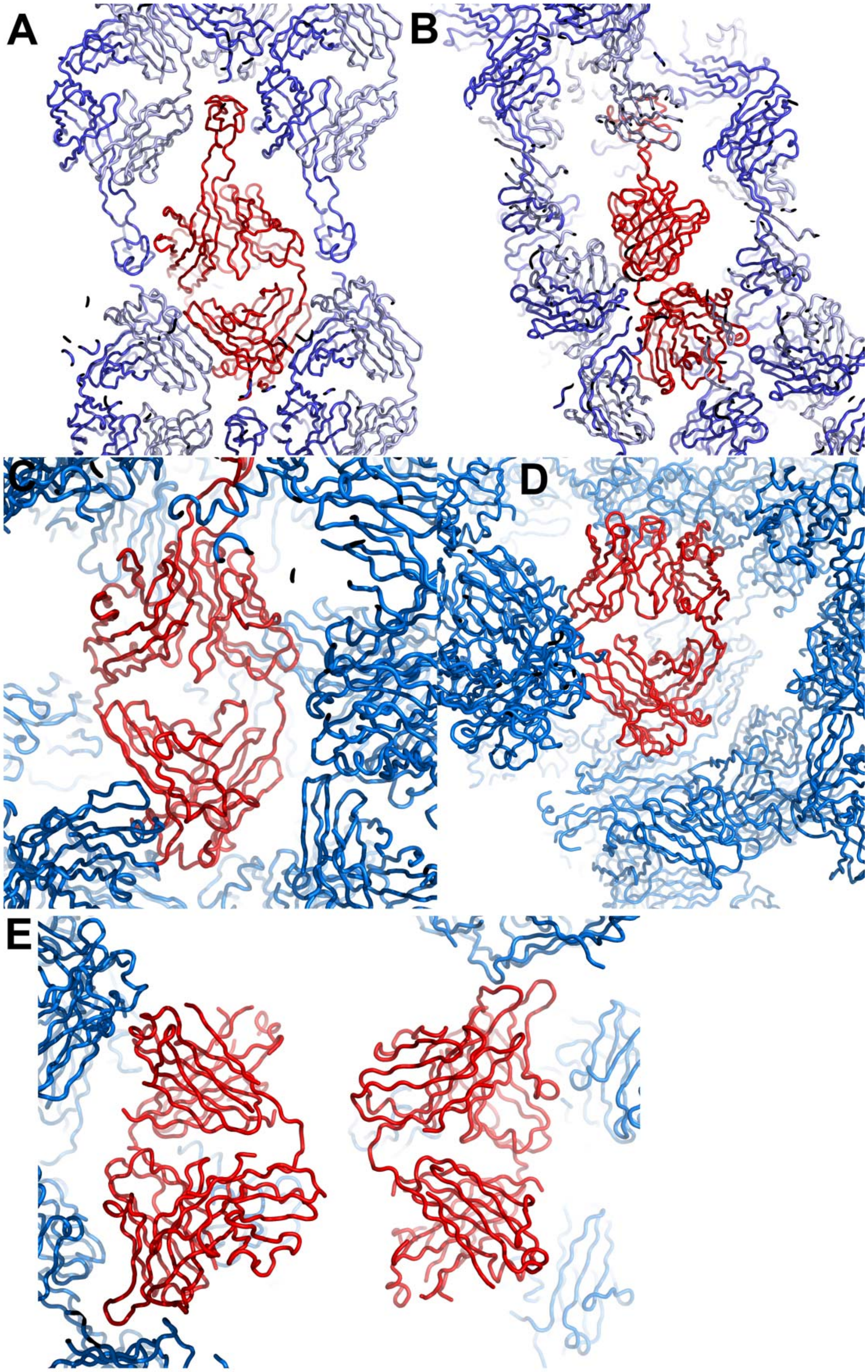

Fig. S7. Crystal packing in NC-Cow1 complex and unliganded structures. (**A,B**) Packing around Fab NC-Cow1 (red) in the unliganded structure. The A and B panels are rotated about 90 degrees. There are no packing contacts to the stalk region but to other parts of the Fab. (**C**) Packing around Fab NC-Cow1 (red) in the complex structure. **(D**) Packing around Fab 35022 (red) in the complex structure. (**E**) Packing around Fab PGT128 (red) in the complex

structure. Two of these Fabs, which are related by a crystallographic two-fold axis, are shown. Although there are some packing contacts to the constant regions, electron density was very poor for all three Fabs in the complex as frequently observed in Fab structures (*2*) and, hence, the constant regions are not included in the final deposited coordinates.

Table S1. X-ray data collection and refinement statistics for Fab NC-Cow1 and quaternary complex

	Fab NC-Cow1	Fab NC-Cow1+ Fab 35022+ Fab PGT128+ BG505 SOSIP.664
**Data Collection**		
Beamline	APS 23-ID-B	APS 23-ID-D
Wavelength (Å)	1.0332	1.0332
Resolution (Å)[a]	28.9 – 2.10 (2.15 – 2.10)	63.9 – 4.08 (4.17 – 4.08)
Space group	C2	R32
Unit cell (Å)	83.06, 69.56, 95.61	215.24, 215.24, 438.80
(°)	90, 106.99, 90	90, 90, 120
Total reflections	125,109 (9203)	285,223 (13,480)
Unique reflections	30,513 (2243)	30,902 (1515)
Multiplicity	4.1 (4.1)	9.2 (8.9)
Completeness (%)	99.9 (99.9)	100.0 (100.0)
Mean (I)/ σ_I	6.0 (1.0)	11.2 (1.2)
R_{merge}[b](%)	14.2 (128)	21.9 (215)
R_{meas}[c](%)	16.2 (146)	23.2 (228)
R_{pim}[d](%)	7.8 (69)	7.6 (76)
$CC_{1/2}$[e](%)	99.1 (30.3)	79.1 (34.0)
**Refinement**		
Refinement resolution (Å)[a]	28.9 – 2.10 (2.17 – 2.10)	48.8 – 4.08 (4.22 – 4.08)
# reflections in refinement (work/free)	28,964 (1543)	29,332 (1556)
R_{work} (%)	20.2 (31.7)	33.3 (43.1)
R_{free} (%)	23.8 (35.3)	34.5 (37.6)
# Protein atoms	3637	10796
# Waters	66	0
# Protein residues	485	1310
RMS (bonds)	0.002	0.004
RMS (angles)	0.58	0.94
Ramachandran favoured, allowed, outliers (%)(*3*)	96.9, 3.1, 0.0	94.0, 5.4, 0.6
Clashscore[f]	0.42	12.3
Wilson B (Å^2)	40	174
Average B (Å^2)	45	213
Protein	45	213
gp120		192
gp41		177
NC-Cow1 Fv		228
35022 Fv		209
PGT128 Fv		273
Solvent	47	NA

[a]Numbers in parentheses are for highest resolution shell

[b]$R_{merge} = \Sigma_{hkl} \Sigma_{i=1,n}|I_i(hkl) - \langle I(hkl)\rangle| / \Sigma_{hkl} \Sigma_{i=1,n} I_i(hkl)$

[c] $R_{meas} = \Sigma_{hkl} \sqrt{(n/n-1)} \, \Sigma_{i=1,n}|I_i(hkl) - <I(hkl)>| / \Sigma_{hkl} \Sigma_{i=1,n} I_i(hkl)$
[d] $R_{pim} = \Sigma_{hkl} \sqrt{(1/n-1)} \, \Sigma_{i=1,n}|I_i(hkl) - <I(hkl)>| / \Sigma_{hkl} \Sigma_{i=1,n} I_i(hkl)$
[e] $CC_{1/2}$ = Pearson Correlation Coefficient between two random half datasets
[f] Number of unfavorable all-atom steric overlaps ≥ 0.4Å per 1000 atoms

Table S2. EM data collection and processing statistics	
**Map**	**BG505 SOSIP.664 + NC-Cow1 (EMD-20500, PDB 6PW6)**
**Data collection**	
Microscope	FEI Talos Arctica
Voltage (kV)	200
Detector	Gatan K2 Summit
Recording mode	Counting
Nominal magnification	36,000
Movie micrograph pixelsize (Å)	1.15
Dose rate (e^-/[(camera pixel)*s])	8.44
Number of frames per movie micrograph	40
Frame exposure time (ms)	250
Movie micrograph exposure time (s)	10
Total dose (e^-/Å^2)	64
Defocus range (μm)	-0.5 to -4.8
**EM data processing**	
Number of movie micrographs	1,506
Number of molecular projection images in map	21,427
Symmetry	C3
Map resolution (FSC 0.143; Å)	4.5
Local resolution range (Å)	4.5-15.0
Map sharpening B-factor (Å^2)	-227
**Structure building and validation**	
Number of atoms in deposited model	
gp120	10,362
gp41	2,913
Fab Fv	1,194
Glycans	1,410
MolProbity score	1.00
Clashscore	1.41
EMRinger score	2.23
RMSD from ideal	
Bond length (Å)	0.02
Bond angles (˚)	1.74
Ramachandran plot	
Favored (%)	97.3
Outliers (%)	0.17
Side chain rotamer outliers (%)	0.56

**Table S3. Molecular surface area buried on BG505 SOSIP.664 by NC-Cow1, CD4, and scFv NIH45-46 and on gp120 by VRC13**						
		Buried molecular surface (Å^2)				
		6OPA	6CM3	5D9Q	4YDJ	
gp120 secondary structure element	gp120 residue	NC-Cow1	CD4	NIH45-46	VRC13	Wu-Kabat variability*
N-terminus	49 GLU	0.0	0.0	17.3	0.0	47.6
β1	96 TRP	0.0	0.0	12.6	21.5	12.1
β1	97 LYS	0.0	0.0	29.2	10.4	15.6
β1	98 ASN	0.0	0.0	5.9	0.0	10.0
β1	99 ASN	0.0	0.0	16.9	0.0	28.4
α1	102 GLU	0.0	0.0	31.9	0.0	16.8
α1	105 HIS	0.0	0.0	12.2	0.0	14.9
α1	109 ILE	0.0	0.0	3.0	0.0	8.2
V1/V2 loop	124 PRO	0.0	32.5	0.0	0.0	11.2
V1/V2 loop	125 LEU	0.0	8.4	0.0	0.0	12.3
V1/V2 loop	126 CYS	0.0	41.2	0.0	0.0	11.0
V1/V2 loop	127 VAL	0.0	0.4	0.0	0.0	9.2
V1/V2 loop	128 THR	0.0	20.1	0.0	0.0	14.7
V1/V2 loop	192 ARG	0.0	23.5	0.0	0.0	19.3
V1/V2 loop	194 ILE	0.0	34.8	0.0	0.0	18.0
V1/V2 loop	196 CYS	0.0	19.0	0.0	0.0	9.0
V1/V2 loop	197 ASN	0.0	0.0	8.8	0.0	12.2
V1/V2 loop	198 THR	0.0	6.3	33.4	0.0	13.3
V1/V2 loop	199 SER	0.0	0.0	12.1	0.0	9.2
B loop	257 THR	0.0	9.0	0.0	0.0	9.0
D loop	275 GLU	1.3	0.0	27.2	17.4	20.7
D loop	276 ASN	0.0	0.0	19.4	0.0	13.3
D loop	278 THR	0.0	3.2	42.3	0.0	20.5
D loop	279 ASN	9.5	34.1	42.3	4.1	32.4
D loop	280 ASN	16.8	34.1	47.7	10.1	15.5
D loop	281 ALA	50.5	52.9	55.7	52.9	26.8
D loop	282 LYS	28.1	23.8	30.9	35.9	16.9
D loop	283 ASN	33.4	26.4	29.6	(Thr)23.6	19.5
V3 loop	327 ARG	0.0	0.0	0.0	11.9	10.2
CD4 binding loop	364 SER	32.7	0.0	5.1	(Pro)15.8	25.1
CD4 binding loop	365 SER	22.4	45.8	47.1	44.1	18.6
CD4 binding loop	366 GLY	35.0	16.2	20.8	45.5	10.1
CD4 binding loop	367 GLY	26.0	25.2	18.2	44.1	8.0
CD4 binding loop	368 ASP	54.3	42.0	35.6	48.4	8.0
CD4 binding loop	369 LEU	2.5	0.0	0.0	57.2	31.2
CD4 binding loop	370 GLU	34.8	16.9	2.5	1.4	11.0
CD4 binding loop	371 VAL	59.2	39.4	35.5	(Ile)44.7	15.2
CD4 binding loop	372 THR	4.8	0.0	0.0	23.8	26.7
CD4 binding loop	373 MET	0.0	0.0	0.0	14.1	32.7

CD4 binding loop	375 SER	2.0	1.8	0.0		21.4
β17	384 TYR	0.0	0.0	0.0	18.2	7.1
β19	419 LYS	0.0	0.0	0.0	29.3	16.9
β19	420 ILE	0.0	0.0	0.0	6.4	9.2
β19	421 LYS	0.0	0.0	0.0	56.5	10.9
β20	423 ILE	0.0	0.0	0.0	26.2	14.6
β20-21	425 ASN	5.3	34.6	11.8	0.0	16.5
β20-21	426 MET	22.2	13.7	0.0	0.0	19.4
β20-21	427 TRP	0.0	29.9	21.6	0.0	11.1
β20-21	428 GLN	0.6	1.6	66.0	0.0	13.3
β20-21	429 ARG	11.2	20.7	0.0	0.0	27.6
β20-21	430 ILE	0.0	90.4	41.4	0.0	11.1
β20-21	431 GLY	0.0	13.3	0.0	0.0	8.1
β20-21	432 GLN	0.0	5.9	0.0	0.0	34.8
β23	455 THR	37.5	25.4	38.9	43.0	17.0
β23	456 ARG	12.9	14.7	19.8	0.0	20.9
β23	457 ASP	18.5	26.3	54.3	2.1	11.1
V5	458 GLY	0.0	33.1	40.4	4.4	19.5
V5	459 GLY	0.0	10.1	38.5	7.3	16.9
V5	460 SER	0.0	9.3	36.9	0.0	53.5
V5	461 THR	0.0	0.0	48.7	0.0	47.8
V5	462 ASN	0.0	0.0	1.9	0.0	40.4
V5	463 SER	0.0	0.0	10.4	0.0	66.4
V5	465 THR	0.0	0.0	18.8	0.0	39.6
V5	466 GLU	0.0	0.0	4.0	0.0	18.7
V5	467 THR	0.0	0.0	27.6	0.0	27.2
V5	469 ARG	46.8	20.6	34.9	20.2	14.2
β24-α5	470 PRO	5.5	0.0	3.1	5.7	10.1
β24-α5	471 GLY	21.1	6.6	7.2	3.4	22.1
β24-α5	472 GLY	4.8	17.8	1.5	6.6	9.0
β24-α5	473 GLY	45.8	23.9	30.9	31.5	7.0
β24-α5	474 ASP	28.2	32.8	39.0	(Asn)23.4	20.8
β24-α5	475 MET	15.4	7.0	9.9	0.0	11.0
β24-α5	476 ARG	4.3	16.5	34.8	(Lys)1.9	21.9
β24-α5	477 ASP	6.2	2.0	5.0	8.6	13.5
β24-α5	480 ARG	0.0	0.0	14.6	11.6	7.1
	neighboring protomer	0.0	0.0	29.5	0.0	
	glycan at N197	115.3	0.0	76.1	0.0	98% conserved
	glycan at N234	0.0	0.0	204.9	0.0	80% conserved
	glycan at N276	19.0	51.2	37.8	0.0	95% conserved
	glycan at N386	0.0	0.0	0.0	65.7	87% conserved

	glycan at N462	0.0	0.0	80.7	0.0	17% conserved
Total buried surface		834	1065	1735	899	
	*Wu-Kabat variability (*4*)= (N*k)/n where N = total number of sequences in alignment, k = number of different amino acid types at given position, and n = number of times the most common amino acid is present at that position. A variability of 1.0 means the residue is 100% conserved.					

Table S4. Molecular surface area buried for different Fab, scFv, or CD4 complexes with Env trimers, gp120 cores, or gp120 outer domains.

						Buried molecular surface ($Å^2$)	
PDB ID	Method/res (Å)	Antibody/CD4	gp120 type	Glycosylation	Trimer state	Fab, scFv or CD4	gp120
6OPA	XRAY	NC-Cow1 Fab	BG505 SOSIP.664	293S/endoH	closed	768	834
5D9Q	XRAY/4.4	NIH45-46 scFv	BG505 SOSIP.664	293S/endoH	closed	1716	1735
5WDU	XRAY/7	NIH45-46 scFv	BG505 SOSIP.664 H72C-H564C	293S/endoH	closed	1449	1409
5FYJ	XRAY/3.4	VRC01 scFv	Clade G X1193.c1SOSIP.664	293S/full	partially open	1606	1560
5FYK	XRAY/3.7	VRC01 scFv	JR-FL	293S/full	closed	1465	1467
6NNF	XRAY/3.5	VRC01 FR-03 scFv	BG505 SOSIP.664	293S/?	closed	1323	1335
5T3X	XRAY/3.9	IOMA Fab	BG505 SOSIP.664	293F/full	closed	1446	1450
5V8L	EM/4.3	3bnc117 Fab Var domain	BG505 SOSIP.664	293F/full	closed	1401	1529
6B0N	XRAY/3.4	PGV19 Fab	BG505 NFL	293F/kif/endoH	closed	1553	1542
6MPG	EM/3.2	VRC03 Fab var domain	BG505 DS SOSIP	293S/full	closed	1559	1539
6OSY	EM/4.3	VRC03 Fab var domain	BG505 DS SOSIP	293S/full	closed	1599	1579
6NM6	XRAY/3.2	N6 FR3-03 scFv*	BG505 SOSIP.664	293S/?	closed	1303	1310
6NNJ	XRAY/3.1	CH31 scFv	BG505 SOSIP.664		closed	1297	1370
6CM3	EM/3.5	CD4	BG505 SOSIp.664 v3.2	293-6E/full	partially open	1014	1065
5VN3	EM/3.7	CD4	B41 SOSIP.664	293F/full	open	1013	1065
2NXY	XRAY/2.0	CD4	HXBc2 core	293S/Swainsonine /endoH	-	927	994
3NGB	XRAY/2.7	VRC01 Fab	Clade A/E 93TH057 core	293S/endoH	-	1001	1016
3U7Y	XRAY/2.4	NIH45-46 Fab	Clade A/E 93TH057 core	Insect cells/endoH	-	1104	1170
4JPV	XRAY/2.8	3bnc117 Fab	Clade A/E 93TH057 core	Not reported; 11 Nags in PDB file	-	1141	1124
3SE8	XRAY/1.9	VRC03 Fab	Clade A/E 93TH057 core	293S/endoH	-	1265	1242
5TE6	XRAY/2.4	N6 Fab	Clade A/E 93TH057 core	293S/endoH	-	1049	1018
5TE7	XRAY/2.2	N6 Fab	Clade C DU172.17 core	293S/endoH	-	1036	988
4JAN	XRAY/3.1	CH103	Clade C ZM176.66 outer domain	293S/endoH	-	662	712
4YE4	XRAY/2.7	HJ16	Clade B HT593.1 core	293S/endoH	-	844	854
4YDJ	XRAY/2.3	VRC13	Clade A/E 93TH057 core	293S/endoH	-	886	899
4YDK	XRAY 2.1	VRC16	Clade A/E 93TH057 core	293S/endoH	-	1088	1049

- N6 FR3-03 has an artificial FR3 region that buries 137$Å^2$ of surface area on a neighboring gp120 subunit, but otherwise should have similar interactions as the wildtype N6

REFERENCES AND NOTES

1. R. Wyatt, P. D. Kwong, E. Desjardins, R. W. Sweet, J. Robinson, W. A. Hendrickson, J. G. Sodroski, The antigenic structure of the HIV gp120 envelope glycoprotein. *Nature* **393**, 705–711 (1998).
2. D. R. Burton, R. C. Desrosiers, R. W. Doms, W. C. Koff, P. D. Kwong, J. P. Moore, G. J. Nabel, J. Sodroski, I. A. Wilson, R. T. Wyatt, HIV vaccine design and the neutralizing antibody problem. *Nat. Immunol.* **5**, 233–236 (2004).
3. P. D. Kwong, J. R. Mascola, HIV-1 vaccines based on antibody identification, B cell ontogeny, and epitope structure. *Immunity* **48**, 855–871 (2018).
4. E. Landais, P. L. Moore, Development of broadly neutralizing antibodies in HIV-1 infected elite neutralizers. *Retrovirology* **15**, 61 (2018).
5. D. Sok, D. R. Burton, Recent progress in broadly neutralizing antibodies to HIV. *Nat. Immunol.* **19**, 1179–1188 (2018).
6. D. R. Burton, J. R. Mascola, Antibody responses to envelope glycoproteins in HIV-1 infection. *Nat. Immunol.* **16**, 571–576 (2015).
7. T. Zhou, K. Xu, Structural features of broadly neutralizing antibodies and rational design of vaccine. *Adv. Exp. Med. Biol.* **1075**, 73–95 (2018).
8. M. Crispin, A. B. Ward, I. A. Wilson, Structure and immune recognition of the HIV glycan shield. *Annu. Rev. Biophys.* **47**, 499–523 (2018).
9. S. S. Saini, B. Allore, R. M. Jacobs, A. Kaushik, Exceptionally long CDR3H region with multiple cysteine residues in functional bovine IgM antibodies. *Eur. J. Immunol.* **29**, 2420–2426 (1999).
10. F. Wang, D. C. Ekiert, I. Ahmad, W. Yu, Y. Zhang, O. Bazirgan, A. Torkamani, T. Raudsepp, W. Mwangi, M. F. Criscitiello, I. A. Wilson, P. G. Schultz, V. V. Smider, Reshaping antibody diversity. *Cell* **153**, 1379–1393 (2013).
11. T. C. Deiss, M. Vadnais, F. Wang, P. L. Chen, A. Torkamani, W. Mwangi, M. P. Lefranc, M. F. Criscitiello, V. V. Smider, Immunogenetic factors driving formation of ultralong VH CDR3 in *Bos taurus* antibodies. *Cell. Mol. Immunol.* **16**, 53–64 (2017).
12. R. L. Stanfield, I. A. Wilson, V. V. Smider, Conservation and diversity in the ultralong third heavy-chain complementarity-determining region of bovine antibodies. *Sci. Immunol.* **1**, aaf7962 (2016).
13. J. Dong, J. A. Finn, P. A. Larsen, T. P. L. Smith, J. E. Crowe Jr., Structural diversity of ultralong CDRH3s in seven bovine antibody heavy chains. *Front. Immunol.* **10**, 558 (2019).
14. R. L. Stanfield, J. Haakenson, T. C. Deiss, M. F. Criscitiello, I. A. Wilson, V. V. Smider, The unusual genetics and biochemistry of bovine immunoglobulins. *Adv. Immunol.* **137**, 135–164 (2018).

15. J. Liljavirta, A. Ekman, J. S. Knight, A. Pernthaner, A. Iivanainen, M. Niku, Activation-induced cytidine deaminase (AID) is strongly expressed in the fetal bovine ileal Peyer's patch and spleen and is associated with expansion of the primary antibody repertoire in the absence of exogenous antigens. *Mucosal Immunol.* **6**, 942–949 (2013).

16. R. W. Sanders, M. J. van Gils, R. Derking, D. Sok, T. J. Ketas, J. A. Burger, G. Ozorowski, A. Cupo, C. Simonich, L. Goo, H. Arendt, H. J. Kim, J. H. Lee, P. Pugach, M. Williams, G. Debnath, B. Moldt, M. J. van Breemen, G. Isik, M. Medina-Ramirez, J. W. Back, W. C. Koff, J. P. Julien, E. G. Rakasz, M. S. Seaman, M. Guttman, K. K. Lee, P. J. Klasse, C. LaBranche, W. R. Schief, I. A. Wilson, J. Overbaugh, D. R. Burton, A. B. Ward, D. C. Montefiori, H. Dean, J. P. Moore, HIV-1 neutralizing antibodies induced by native-like envelope trimers. *Science* **349**, aac4223 (2015).

17. J. van Schooten, M. J. van Gils, HIV-1 immunogens and strategies to drive antibody responses towards neutralization breadth. *Retrovirology* **15**, 74 (2018).

18. D. Sok, K. M. Le, M. Vadnais, K. L. Saye-Francisco, J. G. Jardine, J. L. Torres, Z. T. Berndsen, L. Kong, R. Stanfield, J. Ruiz, A. Ramos, C. H. Liang, P. L. Chen, M. F. Criscitiello, W. Mwangi, I. A. Wilson, A. B. Ward, V. V. Smider, D. R. Burton, Rapid elicitation of broadly neutralizing antibodies to HIV by immunization in cows. *Nature* **548**, 108–111 (2017).

19. R. Pejchal, K. J. Doores, L. M. Walker, R. Khayat, P. S. Huang, S. K. Wang, R. L. Stanfield, J. P. Julien, A. Ramos, M. Crispin, R. Depetris, U. Katpally, A. Marozsan, A. Cupo, S. Maloveste, Y. Liu, R. McBride, Y. Ito, R. W. Sanders, C. Ogohara, J. C. Paulson, T. Feizi, C. N. Scanlan, C. H. Wong, J. P. Moore, W. C. Olson, A. B. Ward, P. Poignard, W. R. Schief, D. R. Burton, I. A. Wilson, A potent and broad neutralizing antibody recognizes and penetrates the HIV glycan shield. *Science* **334**, 1097–1103 (2011).

20. J. Huang, B. H. Kang, M. Pancera, J. H. Lee, T. Tong, Y. Feng, H. Imamichi, I. S. Georgiev, G. Y. Chuang, A. Druz, N. A. Doria-Rose, L. Laub, K. Sliepen, M. J. van Gils, A. T. de la Pena, R. Derking, P. J. Klasse, S. A. Migueles, R. T. Bailer, M. Alam, P. Pugach, B. F. Haynes, R. T. Wyatt, R. W. Sanders, J. M. Binley, A. B. Ward, J. R. Mascola, P. D. Kwong, M. Connors, Broad and potent HIV-1 neutralization by a human antibody that binds the gp41-gp120 interface. *Nature* **515**, 138–142 (2014).

21. L. Kong, J. H. Lee, K. J. Doores, C. D. Murin, J. P. Julien, R. McBride, Y. Liu, A. Marozsan, A. Cupo, P. J. Klasse, S. Hoffenberg, M. Caulfield, C. R. King, Y. Hua, K. M. Le, R. Khayat, M. C. Deller, T. Clayton, H. Tien, T. Feizi, R. W. Sanders, J. C. Paulson, J. P. Moore, R. L. Stanfield, D. R. Burton, A. B. Ward, I. A. Wilson, Supersite of immune vulnerability on the glycosylated face of HIV-1 envelope glycoprotein gp120. *Nat. Struct. Mol. Biol.* **20**, 796–803 (2013).

22. W. B. Struwe, E. Chertova, J. D. Allen, G. E. Seabright, Y. Watanabe, D. J. Harvey, M. Medina-Ramirez, J. D. Roser, R. Smith, D. Westcott, B. F. Keele, J. W. Bess, Jr., R. W. Sanders, J. D. Lifson, J. P. Moore, M. Crispin, Site-specific glycosylation of virion-derived

HIV-1 Env is mimicked by a soluble trimeric immunogen. *Cell Rep.* **24**, 1958–1966.e5 (2018).

23. P. D. Kwong, R. Wyatt, J. Robinson, R. W. Sweet, J. Sodroski, W. A. Hendrickson, Structure of an HIV gp120 envelope glycoprotein in complex with the CD4 receptor and a neutralizing human antibody. *Nature* **393**, 648–659 (1998).

24. T. T. Wu, E. A. Kabat, An analysis of the sequences of the variable regions of Bence Jones proteins and myeloma light chains and their implications for antibody complementarity. *J. Exp. Med.* **132**, 211–250 (1970).

25. D. Corti, J. P. M. Langedijk, A. Hinz, M. S. Seaman, F. Vanzetta, B. M. Fernandez-Rodriguez, C. Silacci, D. Pinna, D. Jarrossay, S. Balla-Jhagjhoorsingh, B. Willems, M. J. Zekveld, H. Dreja, E. O'Sullivan, C. Pade, C. Orkin, S. A. Jeffs, D. C. Montefiori, D. Davis, W. Weissenhorn, Á. McKnight, J. L. Heeney, F. Sallusto, Q. J. Sattentau, R. A. Weiss, A. Lanzavecchia, Analysis of memory B cell responses and isolation of novel monoclonal antibodies with neutralizing breadth from HIV-1-infected individuals. *PLOS One* **5**, e8805 (2010).

26. H.-X. Liao, R. Lynch, T. Zhou, F. Gao, S. M. Alam, S. D. Boyd, A. Z. Fire, K. M. Roskin, C. A. Schramm, Z. Zhang, J. Zhu, L. Shapiro; NISC Comparative Sequencing Program, J. C. Mullikin, S. Gnanakaran, P. Hraber, K. Wiehe, G. Kelsoe, G. Yang, S. M. Xia, D. C. Montefiori, R. Parks, K. E. Lloyd, R. M. Scearce, K. A. Soderberg, M. Cohen, G. Kamanga, M. K. Louder, L. M. Tran, Y. Chen, F. Cai, S. Chen, S. Moquin, X. Du, M. G. Joyce, S. Srivatsan, B. Zhang, A. Zheng, G. M. Shaw, B. H. Hahn, T. B. Kepler, B. T. Korber, P. D. Kwong, J. R. Mascola, B. F. Haynes, Co-evolution of a broadly neutralizing HIV-1 antibody and founder virus. *Nature* **496**, 469–476 (2013).

27. T. Zhou, L. Xu, B. Dey, A. J. Hessell, D. Van Ryk, S. H. Xiang, X. Yang, M. Y. Zhang, M. B. Zwick, J. Arthos, D. R. Burton, D. S. Dimitrov, J. Sodroski, R. Wyatt, G. J. Nabel, P. D. Kwong, Structural definition of a conserved neutralization epitope on HIV-1 gp120. *Nature* **445**, 732–737 (2007).

28. J. Huang, B. H. Kang, E. Ishida, T. Zhou, T. Griesman, Z. Sheng, F. Wu, N. A. Doria-Rose, B. Zhang, K. McKee, S. O'Dell, G. Y. Chuang, A. Druz, I. S. Georgiev, C. A. Schramm, A. Zheng, M. G. Joyce, M. Asokan, A. Ransier, S. Darko, S. A. Migueles, R. T. Bailer, M. K. Louder, S. M. Alam, R. Parks, G. Kelsoe, T. Von Holle, B. F. Haynes, D. C. Douek, V. Hirsch, M. S. Seaman, L. Shapiro, J. R. Mascola, P. D. Kwong, M. Connors, Identification of a CD4-binding-site antibody to HIV that evolved near-pan neutralization breadth. *Immunity* **45**, 1108–1121 (2016).

29. M. F. Flajnik, A cold-blooded view of adaptive immunity. *Nat. Rev. Immunol.* **18**, 438–453 (2018).

30. D. C. Ekiert, A. K. Kashyap, J. Steel, A. Rubrum, G. Bhabha, R. Khayat, J. H. Lee, M. A. Dillon, R. E. O'Neil, A. M. Faynboym, M. Horowitz, L. Horowitz, A. B. Ward, P. Palese, R.

Webby, R. A. Lerner, R. R. Bhatt, I. A. Wilson, Cross-neutralization of influenza A viruses mediated by a single antibody loop. *Nature* **489**, 526–532 (2012).

31. L. E. McCoy, L. Rutten, D. Frampton, I. Anderson, L. Granger, R. Bashford-Rogers, G. Dekkers, N. M. Strokappe, M. S. Seaman, W. Koh, V. Grippo, A. Kliche, T. Verrips, P. Kellam, A. Fassati, R. A. Weiss, Molecular evolution of broadly neutralizing Llama antibodies to the CD4-binding site of HIV-1. *PLOS Pathog.* **10**, e1004552 (2014).

32. M. Lu, X. Ma, L. R. Castillo-Menendez, J. Gorman, N. Alsahafi, U. Ermel, D. S. Terry, M. Chambers, D. Peng, B. Zhang, T. Zhou, N. Reichard, K. Wang, J. R. Grover, B. P. Carman, M. R. Gardner, I. Nikić-Spiegel, A. Sugawara, J. Arthos, E. A. Lemke, A. B. Smith III, M. Farzan, C. Abrams, J. B. Munro, A. B. McDermott, A. Finzi, P. D. Kwong, S. C. Blanchard, J. G. Sodroski, W. Mothes, Associating HIV-1 envelope glycoprotein structures with states on the virus observed by smFRET. *Nature* **568**, 415–419 (2019).

33. A. B. Ward, I. A. Wilson, The HIV-1 envelope glycoprotein structure: Nailing down a moving target. *Immunol. Rev.* **275**, 21–32 (2017).

34. B. M. Stadtmueller, M. D. Bridges, K. M. Dam, M. T. Lerch, K. E. Huey-Tubman, W. L. Hubbell, P. J. Bjorkman, DEER spectroscopy measurements reveal multiple conformations of HIV-1 SOSIP envelopes that show similarities with envelopes on native virions. *Immunity* **49**, 235–246.e4 (2018).

35. A. T. McGuire, S. Hoot, A. M. Dreyer, A. Lippy, A. Stuart, K. W. Cohen, J. Jardine, S. Menis, J. F. Scheid, A. P. West, W. R. Schief, L. Stamatatos, Engineering HIV envelope protein to activate germline B cell receptors of broadly neutralizing anti-CD4 binding site antibodies. *J. Exp. Med.* **210**, 655–663 (2013).

36. A. J. Borst, C. E. Weidle, M. D. Gray, B. Frenz, J. Snijder, M. G. Joyce, I. S. Georgiev, G. B. Stewart-Jones, P. D. Kwong, A. T. McGuire, F. DiMaio, L. Stamatatos, M. Pancera, D. Veesler, Germline VRC01 antibody recognition of a modified clade C HIV-1 envelope trimer and a glycosylated HIV-1 gp120 core. *eLife* **7**, e37688 (2018).

37. J. H. Lee, R. Andrabi, C. Y. Su, A. Yasmeen, J. P. Julien, L. Kong, N. C. Wu, R. McBride, D. Sok, M. Pauthner, C. A. Cottrell, T. Nieusma, C. Blattner, J. C. Paulson, P. J. Klasse, I. A. Wilson, D. R. Burton, A. B. Ward, A broadly neutralizing antibody targets the dynamic HIV envelope trimer apex via a long, rigidified, and anionic β-hairpin structure. *Immunity* **46**, 690–702 (2017).

38. P. D. Kwong, J. R. Mascola, Human antibodies that neutralize HIV-1: Identification, structures, and B cell ontogenies. *Immunity* **37**, 412–425 (2012).

39. T. Zhou, I. Georgiev, X. Wu, Z. Y. Yang, K. Dai, A. Finzi, Y. D. Kwon, J. F. Scheid, W. Shi, L. Xu, Y. Yang, J. Zhu, M. C. Nussenzweig, J. Sodroski, L. Shapiro, G. J. Nabel, J. R. Mascola, P. D. Kwong, Structural basis for broad and potent neutralization of HIV-1 by antibody VRC01. *Science* **329**, 811–817 (2010).

40. X. Wu, Z. Zhang, C. A. Schramm, M. G. Joyce, Y. D. Kwon, T. Zhou, Z. Sheng, B. Zhang, S. O'Dell, K. McKee, I. S. Georgiev, G. Y. Chuang, N. S. Longo, R. M. Lynch, K. O. Saunders, C. Soto, S. Srivatsan, Y. Yang, R. T. Bailer, M. K. Louder; NISC Comparative Sequencing Program, J. C. Mullikin, M. Connors, P. D. Kwong, J. R. Mascola, L. Shapiro, Maturation and diversity of the VRC01-antibody lineage over 15 years of chronic HIV-1 infection. *Cell* **161**, 470–485 (2015).

41. N. A. Doria-Rose, C. A. Schramm, J. Gorman, P. L. Moore, J. N. Bhiman, B. J. DeKosky, M. J. Ernandes, I. S. Georgiev, H. J. Kim, M. Pancera, R. P. Staupe, H. R. Altae-Tran, R. T. Bailer, E. T. Crooks, A. Cupo, A. Druz, N. J. Garrett, K. H. Hoi, R. Kong, M. K. Louder, N. S. Longo, K. McKee, M. Nonyane, S. O'Dell, R. S. Roark, R. S. Rudicell, S. D. Schmidt, D. J. Sheward, C. Soto, C. K. Wibmer, Y. Yang, Z. Zhang; NISC Comparative Sequencing. Program, J. C. Mullikin, J. M. Binley, R. W. Sanders, I. A. Wilson, J. P. Moore, A. B. Ward, G. Georgiou, C. Williamson, S. S. Abdool Karim, L. Morris, P. D. Kwong, L. Shapiro, J. R. Mascola, Developmental pathway for potent V1V2-directed HIV-neutralizing antibodies. *Nature* **509**, 55–62 (2014).

42. C. J. McDaniel, D. M. Cardwell, R. B. Moeller Jr., G. C. Gray, Humans and cattle: A review of bovine zoonoses. *Vector Borne Zoonotic Dis.* **14**, 1–19 (2014).

43. M. E. J. Woolhouse, S. Gowtage-Sequeria, Host range and emerging and reemerging pathogens. *Emerg. Infect. Dis.* **11**, 1842–1847 (2005).

44. R. W. Sanders, R. Derking, A. Cupo, J. P. Julien, A. Yasmeen, N. de Val, H. J. Kim, C. Blattner, A. T. de la Peña, J. Korzun, M. Golabek, K. de Los Reyes, T. J. Ketas, M. J. van Gils, C. R. King, I. A. Wilson, A. B. Ward, P. J. Klasse, J. P. Moore, A next-generation cleaved, soluble HIV-1 Env trimer, BG505 SOSIP.664 gp140, expresses multiple epitopes for broadly neutralizing but not non-neutralizing antibodies. *PLOS Pathog.* **9**, e1003618 (2013).

45. W. Kabsch, *XDS*. *Acta Crystallogr. D Biol. Crystallogr.* **66**, 125–132 (2010).

46. Z. Otwinowski, W. Minor, Processing of X-ray diffraction data collected in oscillation mode. *Methods Enzymol.* **276**, 307–326 (1997).

47. A. J. McCoy, R. W. Grosse-Kunstleve, P. D. Adams, M. D. Winn, L. C. Storoni, R. J. Read, Phaser crystallographic software. *J. Appl. Crystallogr.* **40**, 658–674 (2007).

48. P. Emsley, B. Lohkamp, W. G. Scott, K. Cowtan, Features and development of *Coot*. *Acta Crystallogr. D Biol. Crystallogr.* **66**, 486–501 (2010).

49. P. D. Adams, P. V. Afonine, G. Bunkoczi, V. B. Chen, I. W. Davis, N. Echols, J. J. Headd, L. W. Hung, G. J. Kapral, R. W. Grosse-Kunstleve, A. J. McCoy, N. W. Moriarty, R. Oeffner, R. J. Read, D. C. Richardson, J. S. Richardson, T. C. Terwilliger, P. H. Zwart, PHENIX: A comprehensive Python-based system for macromolecular structure solution. *Acta Crystallogr. D Biol. Crystallogr.* **66**, 213–221 (2010).

50. S. Q. Zheng, E. Palovcak, J. P. Armache, K. A. Verba, Y. Cheng, D. A. Agard, MotionCor2: Anisotropic correction of beam-induced motion for improved cryo-electron microscopy. *Nat. Methods* **14**, 331–332 (2017).

51. K. Zhang, Gctf: Real-time CTF determination and correction. *J. Struct. Biol.* **193**, 1–12 (2016).

52. Z. Berndsen, C. Bowman, H. Jang, A. B. Ward, EMHP: An accurate automated hole masking algorithm for single-particle cryo-EM image processing. *Bioinformatics* **33**, 3824–3826 (2017).

53. D. Kimanius, B. O. Forsberg, S. H. Scheres, E. Lindahl, Accelerated cryo-EM structure determination with parallelisation using GPUs in RELION-2. *eLife* **5**, e18722 (2016).

54. E. F. Pettersen, T. D. Goddard, C. C. Huang, G. S. Couch, D. M. Greenblatt, E. C. Meng, T. E. Ferrin, UCSF Chimera—A visualization system for exploratory research and analysis. *J. Comput. Chem.* **25**, 1605–1612 (2004).

55. R. Y.-R. Wang, Y. Song, B. A. Barad, Y. Cheng, J. S. Fraser, F. DiMaio, Automated structure refinement of macromolecular assemblies from cryo-EM maps using Rosetta. *eLife* **5**, e17219 (2016).

56. V. B. Chen, W. B. Arendall III, J. J. Headd, D. A. Keedy, R. M. Immormino, G. J. Kapral, L. W. Murray, J. S. Richardson, D. C. Richardson, MolProbity: All-atom structure validation for macromolecular crystallography. *Acta Crystallogr. D Biol. Crystallogr.* **66**, 12–21 (2010).

57. B. A. Barad, N. Echols, R. Y.-R Wang, Y. Cheng, F. DiMaio, P. D. Adams, J. S. Fraser, EMRinger: Side chain-directed model and map validation for 3D cryo-electron microscopy. *Nat. Methods* **12**, 943–946 (2015).

58. J. Agirre, J. Iglesias-Fernández, C. Rovira, G. J. Davies, K. S. Wilson, K. D. Cowtan, Privateer: Software for the conformational validation of carbohydrate structures. *Nat. Struct. Mol. Biol.* **22**, 833–834 (2015).

59. M. L. Connolly, The molecular surface package. *J. Mol. Graph.* **11**, 139–141 (1993).

60. M. Li, F. Gao, J. R. Mascola, L. Stamatatos, V. R. Polonis, M. Koutsoukos, G. Voss, P. Goepfert, P. Gilbert, K. M. Greene, M. Bilska, D. L. Kothe, J. F. Salazar-Gonzalez, X. Wei, J. M. Decker, B. H. Hahn, D. C. Montefiori, Human immunodeficiency virus type 1 *env* clones from acute and early subtype B infections for standardized assessments of vaccine-elicited neutralizing antibodies. *J. Virol.* **79**, 10108–10125 (2005).

61. J. K. Haakenson, R. Huang, V. V. Smider, Diversity in the cow ultralong CDR H3 antibody repertoire. *Front. Immunol.* **9**, 1262 (2018).

62. Y. Pasman, A. Kaushik, in *Comparative Immunoglobulin Genetics*, A. Kaushik, Y. Pasman, Eds. (Apple Academic Press, 2014), pp. 187–221.

63. Y. Aida, S. Takeshima, C. L. Baldwin, A. K. Kaushik, in *The Genetics of Cattle,* D. Garrick, A. Ruvinsky, Eds. (CAB International, 2015), pp. 153–191.

64. L. J. Bailey, K. M. Sheehy, P. K. Dominik, W. G. Liang, H. Rui, M. Clark, M. Jaskolowski, Y. Kim, D. Deneka, W. J. Tang, A. A. Kossiakoff, Locking the elbow: Improved antibody Fab fragments as chaperones for structure determination. *J. Mol. Biol.* **430**, 337–347 (2018).
